# Integration of Metabolomics and Transcriptomics for Investigating the Tolerance of Foxtail Millet (*Setaria italica*) to Atrazine Stress

**DOI:** 10.3389/fpls.2022.890550

**Published:** 2022-06-10

**Authors:** Lifang Sun, Libin Liu, Yuting Wang, Yanfei Feng, Wei Yang, Di Wang, Shuren Gao, Xingfen Miao, Wentao Sun

**Affiliations:** ^1^Key Laboratory of Crop Germplasm Improvement and Cultivation in Cold Regions, Key Laboratory of Low Carbon Green Agriculture of Northeast Plain in Ministry of Agriculture and Rural Affairs, Agronomy College of Heilongjiang Bayi Agricultural University, Daqing, China; ^2^Heilongjiang HYHC Company, Daqing, China

**Keywords:** *Setaria italica*, atrazine, metabolomics, transcriptomics, stress

## Abstract

Foxtail millet (*Setaria italica*) is a monotypic species widely planted in China. However, residual atrazine, a commonly used maize herbicide, in soil, is a major abiotic stress to millet. Here, we investigated atrazine tolerance in millet based on the field experiments, then obtained an atrazine-resistant variety (Gongai2, GA2) and an atrazine-sensitive variety (Longgu31, LG31). To examine the effects of atrazine on genes and metabolites in millet plants, we compared the transcriptomic and metabolomic profiles between GA2 and LG31 seedling leaves. The results showed that 2,208 differentially expressed genes (DEGs; 501 upregulated, 1,707 downregulated) and 192 differentially expressed metabolites (DEMs; 82 upregulated, 110 downregulate) were identified in atrazine-treated GA2, while in atrazine-treated LG31, 1,773 DEGs (761 upregulated, 1,012 downregulated) and 215 DEMs (95 upregulated, 120 downregulated) were identified. The bioinformatics analysis of DEGs and DEMs showed that many biosynthetic metabolism pathways were significantly enriched in GA2 and LG31, such as glutathione metabolism (oxiglutatione, γ-glutamylcysteine; *GSTU6*, *GSTU1*, *GSTF1*), amino acid biosynthesis (L-cysteine, N-acetyl-L-glutamic acid; *ArgB*, *GS*, *hisC*, *POX1*), and phenylpropanoid biosynthesis [*trans-*5-o-(4-coumaroyl)shikimate; *HST*, *C3′H*]. Meanwhile, the co-expression analysis indicated that GA2 plants had enhanced atrazine tolerance owing to improved glutathione metabolism and proline biosynthesis, and the enrichment of scopoletin may help LG31 plants resist atrazine stress. Herein, we screened an atrazine-resistant millet variety and generated valuable information that may deepen our understanding of the complex molecular mechanism underlying the response to atrazine stress in millet.

## Introduction

Atrazine (2-chloro-4-ethylamino-6-isopropylamino-1,3,5-triazine) is a synthetic herbicide that has been extensively applied for the control of broadleaf and grass weeds in field-planted sorghum, wheat, and maize (Gast, 1970; Lebaron et al., 2011; Daniele and Maggi, 2017; Felisbino et al., 2018). However, atrazine residue can remain for up to 231 days in the environment (Karlsson et al., 2016), and previous studies have shown that increased atrazine concentrations in the soil are related to exponentially increased accumulated rainfall in the soil. These studies showed that under the most severe drought conditions, injury to cereal crops could occur up to 3 years after atrazine application at 1.2–1.5 kg/ha (Moyer and Blackshaw, 1993). In addition, the application of atrazine over years may lead to its accumulation in the soil, which not only damages plants but also harms the health of humans, animals, and aquatic organisms (Contreras et al., 2020). Therefore, the toxicity of atrazine and its implications have consequently been the focus of considerable attention.

The mechanisms of toxicity of atrazine to plants have been studied, and it appears that atrazine can cause oxidative damage to plants and decrease the efficiency of photosynthesis. Erinle et al. (2018) found that the levels of malondialdehyde (MDA) and reactive oxygen species (ROS) in pearl millet significantly increased with increasing atrazine concentrations in the soil, whereas antioxidant gene expression was significantly suppressed, reaching its lowest when atrazine was applied at 50 mg/kg. The content of thiobarbituric acid reactive substances in the roots of pearl millet exposed to atrazine for 6 days increased 26% compared with the treatment that was exposed for 2 days, and the ROS also increased (Zhang Y. et al., 2021). The activities of antioxidases in the leaf and root of *Pennisetum americanum* L. were increased to protect the plant from atrazine-induced oxidative damage (Jiang et al., 2016). In addition, atrazine can suppress electron transport at both the donor and receptor sides and acts on the absorption, transfer, and utilization of light energy by damaging the photosystem II reaction center of the microalga *Chlorella* sp. (Sun C. et al., 2020). The effective quantum yield of photosystem II and the electron transport rate of photosynthesis in *Handroanthus heptaphyllus* (Vell.) were gradually reduced by the action of atrazine (Barbosa et al., 2021). These studies suggest that the mechanism of atrazine toxicity is complex and depends on numerous molecular changes.

Metabolomics, as a new discipline, can well analyze all low molecular weight metabolites in a given organism or tissue (Goodacre et al., 2004). Based on the Liquid Chromatography-Mass Spectrometer (LC-MS) and/or Gas Chromatography-Mass Spectrometer (GC-MS) technologies, it has been widely performed to identify and assess the expression levels of multiple metabolites in various studies, all of which obtained valuable results in areas such as human diseases (Zhang K. H. et al., 2021), plant nutrition (Cao et al., 2020), plant microorganism (Skliros et al., 2018), and plant development (Zhang et al., 2019). In addition, high-throughput sequencing technology (RNA-Seq) has become an important tool for transcriptome analysis to detect differentially expressed genes among samples (Shan et al., 2021). Recently, metabolomics combined with transcriptome sequencing has provided a powerful way to study the metabolic phenotype of organisms and related genes for further understanding the molecular and biological mechanisms of functional genomics (Li et al., 2018; Sun J. H. et al., 2020; Zhang et al., 2020).

Foxtail millet (*Setaria italica* L.) is an ancient crop that is widely planted in China because it is rich in protein and crude fat. However, millet is sensitive to herbicides, thereby creating the need for manual weeding. The continuous planting of maize leads to the accumulation of atrazine residue in the soil, as is apparent in the Heilongjiang province of China, and seriously affects the cultivation of millet as a rotation crop. Therefore, the aims of this study were to screen atrazine resistant millet varieties and to evaluate the expression of specific genes and metabolites at the seedling stage using transcriptomics and metabolomics profiles. These data will be valuable for providing basic information to understand the potential biological processes that are relevant to the mechanism of atrazine stress on millet plants.

## Materials and Methods

### Plant Materials and Atrazine Treatment in Field

Based on the stress results of atrazine in the germination stage by Wang et al. (2021), 20 different resistant millet varieties, widely planted in China, were selected for field experiments to further verify their responses to atrazine (Table 1). The experiment was conducted in 2019 at the 5th management zone of Yinlang Farm (124° 44′ 13.89″ E, 56° 29′ 53.60″ N) in Daqing City, Heilongjiang Province, China. This site had an average annual temperature of 4.6°C, an active accumulated temperature of >2800°C, annual average sunshine of 2782.5 h, and a frost-free period of 136 days.

**TABLE 1:** Varieties used in the field experiment.

No.	Varieties	Response to atrazine	No.	Varieties	Response to atrazine
1	Jingu 26 (JG26)	MR	11	Nenxuan 15 (NX15)	HS
2	Fenxuan 3 (FX3)	HR	12	Longgu 25 (LG25)	HS
3	Zigengu (ZGG)	HR	13	Zuantoubai (ZTB)	MS
4	Jingu 40 (JG40)	MR	14	Jinsuihuangjin (JSHJ)	HS
5	Dabaigu (DBG)	MR	15	Longgu 31 (LG31)	HS
6	Gongai 2 (GA2)	HR	16	Huangjindungu (HJDG)	MS
7	Shandongliushan (SDLS)	MR	17	Gongai 5 (GA5)	MS
8	Shanxiwugu (SXWG)	HR	18	Yangu 2 (YG2)	HS
9	Hebeixianggu (HBXG)	HR	19	Zigenbai (ZGB)	MS
10	Caogu (CG)	HR	20	Baigenhongniangu (BGHNG)	MS

*HR, high resistant; MR, middle resistant; MS, middle sensitive; HS, high sensitive.*

The experiment was conducted using a completely randomized design with three replications. The plot size was 9.75 m^2^, with 5-m rows, 0.65-m row spacing, and 3 lines per repeat. The sowing density was 600000 plants/hm^2^. Following the experimental screening results of atrazine stress in the germination stage by Wang et al. (2021), a mixture of approximately 13.55 ml of 38% fully mixed atrazine suspension and 800 ml of water (approximately 11 μmol/L) was sprayed evenly on each plot after sowing for 1 day. Water was used as the control. Additionally, 37.5 kg/hm^2^ urea, 225 kg/hm^2^ diammonium phosphate, and 120 kg/hm^2^ potassium sulfate were applied as base fertilizers, after which approximately 120 kg/hm^2^ urea was applied at the heading stage. Jinan Tianbang Chemical Co., Ltd., Jinan, Shandong province, China, provided the 38% atrazine suspension.

### Determination Indicators and Methods

At the 3-leaf seedling stage, fresh leaf tissues were used to determine the physiological and biochemical indexes, including the chlorophyll content through ethanol extraction and colorimetric methods described by Liu et al. (2020), malondialdehyde (MDA) content using the thiobarbituric acid method as described by Akbulut and Yigit (2010), and soluble protein content using the coomassie brilliant blue G-250-binding method as described by Bradford (1976), with some modifications. Briefly, the whole plant at the 3-leaf seedling stage was transferred into paper bags, heated at 105°C for 30 min, then dried at 80°C to constant weight, and weighed using a 1/10000 gram balance with three biological repetitions. Subsequently, the average was taken after each determination.

At the 7–8-leaf seedling stage, fully expanded young leaves of 3 plants with the same growth pattern were selected for each treatment. Subsequently, we measured net photosynthetic rates, transpiration rates, and stomatal conductance of their inverter clovers using a LI-COR 6400 portable photosynthetic instrument (LI-COR Inc., Lincoln, NE, United States) on sunny days (9:00 to 11:00 h) at 1500 μmol m^–2^ s^–1^ PPFD. Ambient water vapor pressure, CO_2_ concentration, and leaf temperature were maintained at 1.2 ± 0.1 kPa, 400 μmol m^–2^ s^–1^, and 25 ± 1.0°C, respectively. The average values were taken for data analysis.

At maturity, five panicles were randomly selected from each treatment to determine the grain weight per panicle. The average value was taken for data analysis.

### Statistical Analyses

All measurements conducted were repeated at least in triplicate. Relative values between the treatment and control were used in the data analysis through repetitions to avoid differences in genotypes. Data analysis was conducted using SPSS-26.0 (SPSS, Chicago, IL, United States) to determine the weight value of each indicator through principal component analysis (PCA), as described by Dong et al. (2016). The standardized principal component (PC) scores were extracted from the correlation matrix, and those PCs with eigenvalues of ≥1.00 were selected. It should be emphasized that to make the trend of each index consistent, the relative value of MDA should be converted to its reciprocal. Ultimately, the subordinate function method was used to comprehensively evaluate the resistance of different millet genotypes to stress.

### RNA Sequencing, Annotation, and Expression Analysis

#### Material Preparation

All seeds of the resistant and tolerant millet varieties were sterilized in 0.1% NaClO (v/v) and sown in germination boxes (20-cm diameter and 15-cm height; 5 holes and 5 plants per hole) containing approximately 0.5 kg of soil, flint, and perlite in a 3:2:1 ratio. We set up two treatments [control (water) and atrazine] in four boxes each. The concentration of atrazine was consistent with the germination stage, according to Wang et al. (2021). Subsequently, all pots were placed in a greenhouse (25 ± 1.5°C during the day and 20 ± 1.5°C at night) with a photoperiod of 14:10 h (light/dark). These pots were watered using 1/2 concentration of the Hoagland nutrient solution once a day. At the 3-leaf seedling stage, the leaves were harvested and stored at −80°C for further analysis.

#### RNA Extraction

A total of 12 samples (3 biological repetitions per treatment group of each variety) were used for transcriptome sequencing. Total RNA was extracted from leaves using the RNAiso Reagent Plant Kit (Takara, Dalian, China) (Sun et al., 2012) following the manufacturer’s protocols. The quality and quantity of RNA were assessed using a NanoDrop 1000 spectrophotometer and Agilent 2100 bio-analyzer before constructing a cDNA library at Beijing Biomarker Biotechnology Co., Ltd., Beijing, China^1^.

#### cDNA Library Construction and Illumina Sequencing

Total RNA was treated with DNase I, following which oligo(dT) magnetic beads were used to enrich Poly (A+) RNA for digestion using RNA Fragmentation buffer. The first-strand cDNAs were synthesized using the mRNA fragments as templates with random hexamer primers. Subsequently, the second strands were synthesized using dNTPs, DNA polymerase I, and RNase H; purified using QIAquick PCR purification kit (Qiagen, Hilden, Germany); and then resolved into EB buffer for end reparation and single nucleotide A (adenine) addition. The short fragments were then connected with adapters. Fragments suitable for PCR amplification were selected through agarose gel electrophoresis. During qPCR, the quantity and quality of the sample library were assessed using Agilent 2100 bio-analyzer and ABI StepOnePlus Real-Time PCR Systems. Subsequently, the library was sequenced using Illumina HiSeq 4000 or other sequencers, as necessary, at Beijing Biomarker Biotechnology Co., Ltd. (see text footnote 1).

#### *De novo* Assembly and Data Analysis

After sequencing, the raw reads were filtered to remove low-quality, adaptor-polluted, and >10% unknown base (N) reads to obtain clean reads. The clean reads of each library were then mapped to a reference genome^2^ using the HISAT2 method (Kim et al., 2015). The unique mapped reads were assembled *de novo* to obtain reconstructed transcripts using StringTie (Pertea et al., 2015) after genome mapping. The novel transcripts in our samples were identified by comparing the reconstructed transcripts with genome annotation information according to cuffcompare, a tool of cufflinks (Trapnell et al., 2012). Subsequently, the coding potentials of novel transcripts were predicted using CPC methods (Kong et al., 2007) to merge with reference transcripts for obtaining a complete reference for further analysis.

#### Gene Expression Level Analysis

The gene expression level of each sample was calculated based on the expected number of fragments per kilobase of transcript sequence per millions fragments mapped after mapping the clean reads to the reference genome using Bowtie 2 (Langmead and Salzberg, 2012). The differentially expressed genes (DEGs) between the two samples were also analyzed using DESeq two methods with adjusted *p*-values. The DEGs were identified using the following parameters: false discovery rate (FDR) of <0.01 and absolute fold change of ≥2. Gene Ontology (GO) and Kyoto Encyclopedia of Genes and Genomes (KEGG) pathway enrichment analyses of DEGs were further performed based on the major public database (Kanehisa, 2008).

### Ultra-Performance Liquid Chromatography/Tandem Mass Spectrometry-Based Metabolomics Analysis

#### Metabolite Extraction

Fully ground tissue samples were first dissolved in extract solution (volume ratio of methanol to acetonitrile = 1:1, internal standard concentration 2 mg/L). Then, the samples were treated with ultrasound for 10 min in an ice bath, followed by storage at −20°C for 1 h and centrifugation at 12000 × *g* for 15 min at 4°C. Next, 500 μl of the supernatant was transferred into a new 1.5-ml tube for drying, followed by the addition of 160 μl of extract solution (volume ratio of acetonitrile to water = 1:1). The solution was vortexed for 30 s and subjected to ultrasound for 10 min in an ice bath for redissolution. Afterward, the suspension was allowed to stand for 30 min at 4°C before centrifugation (12000 × *g* at 4°C for 15 min) to obtain supernatants ready for injection. This yielded 12 samples (4 treatment groups, including GCK, GT, LCK, and LT, each with 3 biological replicates) using the same method. A quality control (QC) sample was prepared by combining equal aliquots from each sample. All samples were stored at 4°C for analyses within 3 days.

#### Ultra-Performance Liquid Chromatography/Tandem Mass Spectrometry Analysis

Millet extracts were analyzed using an ACQUITY UPLC I-Class PLUS system (Waters Corporation, Milford, MA, United States) coupled to a Xevo G2-XS QTof system (Waters Corporation, Milford, MA, United States) with an electrospray ionization (ESI) source under positive and negative modes. In the ESI+ and ESI− modes, mobile phases A and B were 0.1% formic acid aqueous solution and 0.1% formic acid acetonitrile, respectively. The injection volume was 1 μL. Chromatographic separation was achieved using an ACQUITY UPLC HSS T3 column (2.1 mm × 100 mm, 1.8 μm; Waters Pacific Pte. Ltd, Milford, MA, United States) at a flow rate of 400 μL/min using the following gradient: 98% A and 2% B for 0.25 min, decreased to 2% A and increased to 98% B within 13 min, and then increased to 98% A and decreased to 2% B in the last 2 min.

A Waters Xevo G2-XS QTOF high-resolution mass spectrometer can collect primary and secondary mass spectrometry data in the MSe mode under the control of the acquisition software (MassLynx V4.2, Waters Corporation, Milford, MA, United States). In each data acquisition cycle, it can simultaneously conduct dual-channel data acquisition for low and high collision energies. The low collision energy was 2 V, the high collision energy range was 10–40 V, and the scanning frequency was 0.2 s.

#### Data Preprocessing and Analysis

The collected raw data were processed using the Progenesis QI software for peak extraction, peak alignment, and other data processing operations. Metabolite quantification and theoretical fragment identification were conducted based on the online METLIN^3^ database of the Progenesis QI software and self-built library of Beijing Biomarker Biotechnology Co., Ltd. Theoretical fragment identification parameters and mass deviation were all within 100 ppm.

The follow-up analysis was conducted after normalizing the original peak area information with the total peak area. PCA and Spearman’s correlation analysis were performed to assess the repeatability of the test and quality control samples. According to the grouping information, we calculated and compared different multiples. First, the *t*-test was used to assess significant differences (*p*-value) between the compounds. The variable importance in the projection (VIP) value of the model was calculated using multiple cross validation. The differentially expressed metabolites (DEMs) were identified using the following screening criteria: absolute fold change (FC) of ≥1.5 and VIP of ≥1. The DEMs were queried to classify pathway information based on KEGG and human metabolome databases (HMDB). The extraction, identification, and quantification of metabolites were conducted at Beijing Biomarker Biotechnology Co., Ltd. (see text footnote 1).

### Coexpression Network Analysis of Transcriptome and Metabolome

According to the fold changes of each DEG and DEM, the Pearson’s correlation coefficient was calculated. Before calculating the correlation, the data were preprocessed using the *Z*-value transformation method. Correlations corresponding to a coefficient of *R*^2^ > 0.8 and *p*-value of <0.05 were selected. The relationship between the metabolome and transcriptome was illustrated using BMKCloud (see text footnote 1).

### Real-Time Quantitative Reverse Transcription PCR Validation of Differentially Expressed Genes

The leaves of the millet plant were used for real-time quantitative reverse transcription PCR (qRT-PCR). The method used followed the procedures described in a previous report (Sun et al., 2018). The primers were designed and synthesized according to the gene sequences in the millet sequence database (see text footnote 2) and the 25S RNA sequence was used as the endogenous control (Supplementary Table 1). At the end of the PCR for each sample, based on the fluorescence logarithmic graph, the appropriate threshold was chosen and the Ct values were obtained to analyze the transcript levels of each gene using the 2^–ΔΔCt^ method (Livak and Schmittgen, 2001). The Bio-Rad CFX96 Real-Time System (Bio-Rad, Hercules, CA, United States) and CFX Manager System software version 2.0 were used in following the manufacturer’s instructions in this study.

## Results

### Acquisition of Resistant/Tolerant Millet Varieties Under Atrazine Stress

To analyze the sensitivity of different millet varieties to atrazine, eight indicators in different growth stages were detected and the data were subjected to PC factor analysis. Kaiser–Meyer–Olkin (KMO) and Bartlett’s ball tests were conducted on the relative value data of each trait using SPSS (Supplementary Table 2). The results showed that the KMO test value was 0.819 > 0.5 and the probability of significance in Bartlett’s ball test was *p* < 0.05, indicating that the test data conformed to spherical distribution (Table 2). Therefore, the experimental data of this study were suitable for factor analysis.

**TABLE 2:** Kaiser–Meyer–Olkin (KMO) and Bartlett’s test.

Kaiser–Meyer–Olkin measure of sampling adequacy		0.819
Bartlett’s test of sphericity	Approx. chi-square	58.013
	*df*	28
	Sig.	0.001

In Table 3, the eigenvalues of the first three PCs were >1, and the cumulative contribution rate reached 76.722%, reflecting most of the information of the 8 indexes. Therefore, the first three PCs were selected to evaluate the eight indexes of atrazine tolerance in millet. The confidence level of the evaluation was 76.722%. Based on PCA, the weight value of each indicator was calculated. Results are shown in Table 3. Ultimately, the comprehensive values of atrazine tolerance in 20 foxtail millet varieties were calculated (Table 4). This result showed that the *T* value of Gongai 2 (GA2) was the highest (0.91), whereas that of Longgu 31 (LG31) was the lowest (0.09), indicating that GA2 was not sensitive to atrazine, whereas LG31 was sensitive to atrazine. Therefore, GA2 and LG31 varieties were used further analysis.

**TABLE 3:** First three principal components (PCs) of variable project, eigenvalues, variability (%), cumulative (%), and weight value.

Variable	PC1	PC2	PC3	Weight value
MDA	0.605	−0.382	0.433	0.10
Soluble protein	0.157	0.814	0.471	0.14
Chlorophyll content	0.817	−0.126	−0.299	0.10
Net photosynthetic rate	0.803	−0.070	−0.303	0.10
Transpiration rate	0.752	0.348	−0.046	0.15
Stomatal conductance	0.832	0.220	0.059	0.16
Dry matter	0.880	0.009	−0.161	0.15
Grain weight per panicle	0.440	−0.430	0.634	0.09
Eigenvalue	3.929	1.184	1.025	
Variance contribution (%)	49.111	14.804	12.807	
Cumulative variance contribution (%)	49. 111	63.915	76.722	

**TABLE 4:** Comprehensive evaluation of foxtail millet under atrazine stress.

Varieties	MDA	Soluble pro.	Chlorophyll content	Net photosynthetic rate	Transpiration rate	Stomatal conductance	Dry matter	Grain weight per panicle	*T* value	Ranking
JG26	0.99	0.35	0.75	0.72	0.26	0.28	0.88	0.85	0.59	7
FX3	0.67	0.19	0.99	1.00	1.00	0.91	0.95	0.73	0.80	2
ZGG	0.21	0.06	0.46	0.34	0.31	0.35	0.34	0.48	0.31	16
JG40	0.67	0.37	0.35	0.13	0.18	0.41	0.23	0.49	0.30	17
DBG	0.00	0.53	0.87	0.58	0.59	0.47	0.56	0.52	0.52	10
GA2	0.99	0.58	0.99	0.99	0.99	1.00	0.92	0.84	0.91	1
SDLS	0.89	0.30	0.89	0.62	0.73	0.90	0.86	0.78	0.74	4
SXWG	0.17	0.80	0.39	0.65	0.46	0.82	0.70	0.00	0.54	9
HBXG	0.05	0.59	0.52	0.79	0.94	0.51	0.78	0.56	0.62	5
CG	0.66	0.43	0.80	0.95	0.70	1.02	1.00	0.85	0.80	3
NX15	0.28	0.61	0.13	0.45	0.11	0.25	0.00	1.00	0.32	14
LG25	0.31	0.00	0.20	0.17	0.52	0.35	0.04	0.54	0.26	19
ZTB	0.48	1.00	0.17	0.20	0.72	0.78	0.34	0.78	0.59	6
JSHJ	0.34	0.22	0.06	0.63	0.43	0.28	0.09	0.53	0.31	15
LG31	0.15	0.08	0.00	0.00	0.00	0.07	0.16	0.30	0.09	20
HJDG	0.77	0.23	0.63	0.86	0.40	0.90	0.37	0.35	0.56	8
GA5	0.29	0.49	0.60	0.69	0.57	0.37	0.29	0.36	0.45	12
YG2	0.19	0.16	0.88	0.83	0.22	0.74	0.54	0.74	0.52	11
ZGB	0.51	0.16	0.13	0.65	0.42	0.46	0.45	0.80	0.43	13
BGHNG	0.27	0.28	0.60	0.12	0.20	0.00	0.42	0.43	0.27	18

*T is the comprehensive value of atrazine tolerance in different varieties.*

### Transcriptome Analysis of Millet Under Atrazine Stress

**FIGURE 1:**
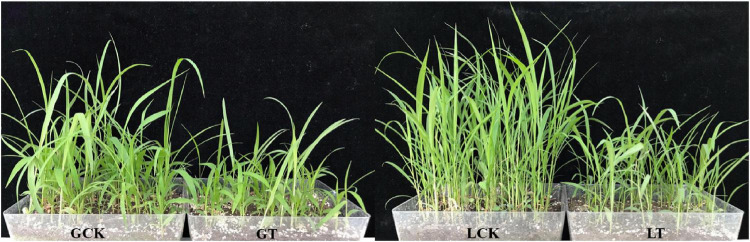
Phenotypes of resistant/sensitive millet under atrazine stress at the seedling stage. GCK, GA2 (Gongai 2) treated with water; GT, GA2 treated with atrazine; LCK, LG31 (Longgu 31) treated with water; LT, LG31 treated with atrazine.

For a deeper understanding of the molecular mechanisms occurring in the millet plant under atrazine stress, its leaves (Figure 1) were used for RNA-Seq, and the raw data of RNA-Seq were uploaded to the NCBI website. With three biological replicates, a total of 87.16 Gb of clean data with 94.31% of bases scoring Q30 were obtained. Of the total clean reads, 93.54–94.91% were unique matches with the *Setaria italic* L. reference genome and 35,747 unique genes were expressed in the millet leaves, of which 1,163 were novel genes and only 856 genes were successfully annotated (Supplementary Table 3).

The expression levels of the transcripts for each sample were calculated to analyze the different resistant and sensitive millet varieties. A total of 3,981 DEGs were identified in the samples of both varieties: 2,208 (501 upregulated and 1,707 downregulated) genes in the Gongai variety and 1,773 (761 upregulated and 1,012 downregulated) genes in the Longgu variety (FC ≥ 2 and FDR < 0.01; Table 5). The detailed information of the genes is presented in Supplementary Table 4. The results also showed that the number of downregulated genes (1,707 in GA2 and 1,012 in LG31) was significantly higher than the number of upregulated genes (501 in GA2 and 761 in LG31) under atrazine stress. The results demonstrated that atrazine could induce changes in transcript levels in millet leaves and that the expression of most genes may be inhibited to adapt to atrazine stress.

**TABLE 5:** DEGs under atrazine stress in millet leaves.

Samples	Total of DEGs	Up	Down
GCK and GT	2208	501	1707
LCK and LT	1773	761	1012

### Functional Analysis of Differentially Expressed Genes in Millet Under Atrazine Stress

**FIGURE 2:**
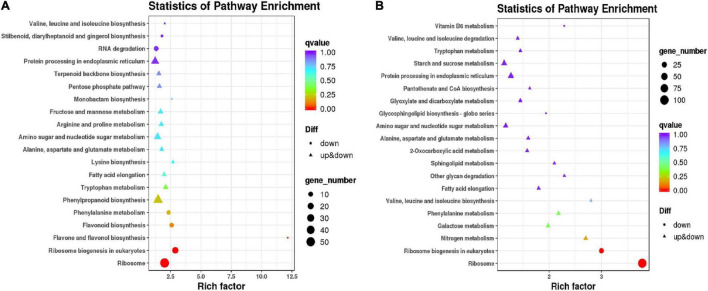
Venn diagram and KEGG analysis of differentially expressed genes in resistant/sensitive millet. Venn diagram of DEGs between two samples: **(A)** GCK and GT; **(B)** LCK and LT.

To classify the functions of DEGs, GO, and KEGG analyses were performed to clarify the biological pathways and functions in millet leaves affected by atrazine. The results showed that the significantly enriched GO categories were “metabolic process,” “cellular process,” and “single-organism process” in the resistant and sensitive millet varieties (Supplementary Figure 1). However, the KEGG analysis results implied that the resistant and sensitive millet varieties had different metabolic pathways (Supplementary Table 4). Based on the results presented in Figure 2A, when comparing GT with GCK, the DEGs enriched in “phenylalanine biosynthesis,” “flavonoid biosynthesis,” “flavone and flavonol biosynthesis,” “ribosome biogenesis,” and “ribosome” showed remarkable downregulation, whereas those enriched in “lysine biosynthesis,” “arginine and proline metabolism,” “fructose and mannose metabolism,” “pentose phosphate pathway,” and “terpenoid backbone biosynthesis” were significantly upregulated. In the “LCK vs. LT” comparison, except for the “ribosome,” “ribosome biogenesis in eukaryotes,” and “vitamin B6 metabolism” pathways, the other metabolic pathways with DEGs were significantly upregulated (Figure 2B).

### Metabolome Analysis of Millet Under Atrazine Stress

**FIGURE 3:**
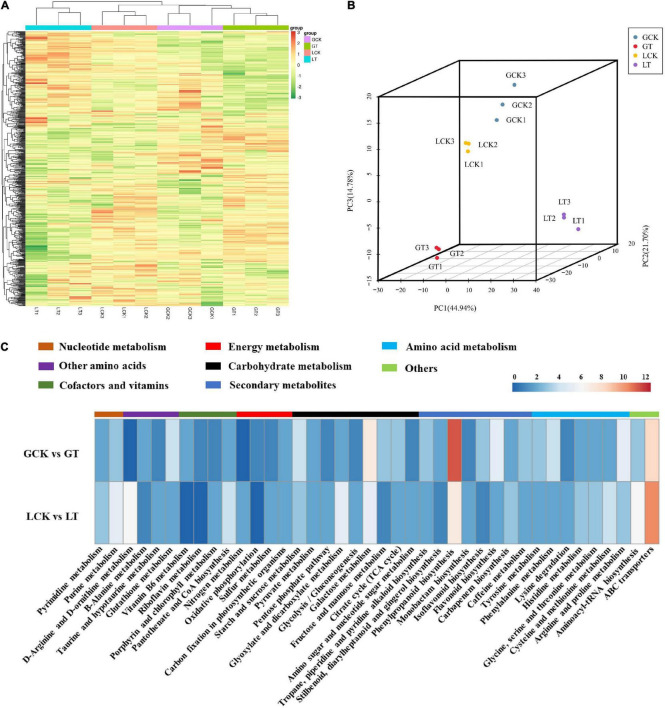
Metabolite analysis using the leaves of resistant/sensitive millet varieties under atrazine stress. **(A)** Heat map visualization of metabolites. Each metabolite is represented by a single row and each example is visualized in a single column; red indicates upregulation and green indicates downregulation. **(B)** PCA of metabolites. PC1, the first principal component; PC2, the second principal component; PC3, the third principal component. **(C)** Distribution of pathways of DEMs annotated in the KEGG data library of resistant/sensitive (GA2/LG31) millet varieties.

For an overview of the metabolomic changes induced by atrazine treatment in millet, the widely targeted metabolomics was analyzed using LC–MS/MS. A total of 636 metabolites were obtained from all samples (Figure 3A and Supplementary Table 5) and divided into four groups according to PCA (Figure 3B). On the basis of FC of ≥1.5, the numbers of upregulated and downregulated metabolites in GCK and GT and LCK and LT were 82 and 95 and 110 and 120, respectively (Supplementary Table 5). Moreover, these DEMs were identified into 65 species and were mainly involved in phenylpropanoid biosynthesis (18), biosynthesis of secondary metabolites (59), metabolic pathways (82), glutathione metabolism (4), flavonoid biosynthesis (7), carbon metabolism (13), arginine and proline metabolism (8), glutathione metabolism (4), glyoxylate and dicarboxylate metabolism (7), purine metabolism (8), and others (Figure 3C and Supplementary Table 6). The DEMs related to proline metabolism, glutathione metabolism, and flavonoid metabolism were significantly accumulated in GCK and GT and LCK and LT. The results implied that atrazine can induce changes in antioxidant levels in millet leaves.

### Relationship Analysis Between Differentially Expressed Genes and Differentially Expressed Metabolites in Millet Under Atrazine Stress

To analyze the relationship between DEGs and DEMs under atrazine stress in millet, a coexpression network analysis of DEGs and DEMs was performed (Pearson’s coefficient *R*^2^ > 0.8 and *p* < 0.05; Supplementary Table 7). The results of the KEGG analysis between the DEGs and DEMs are presented in Supplementary Figure 2. The results implied that GA2 and LG31 millet varieties have different regulation mechanisms under atrazine stress. For example, the DEGs and DEMs in GCK vs. GT were mainly involved in flavonoid biosynthesis, glutathione metabolism, amino acid biosynthesis, and fructose/mannose metabolism (Supplementary Figure 2A). However, in LCK vs. LT, the galactose, starch and sucrose, and thiamine metabolisms were mainly enriched (Supplementary Figure 2B).

**FIGURE 4:**
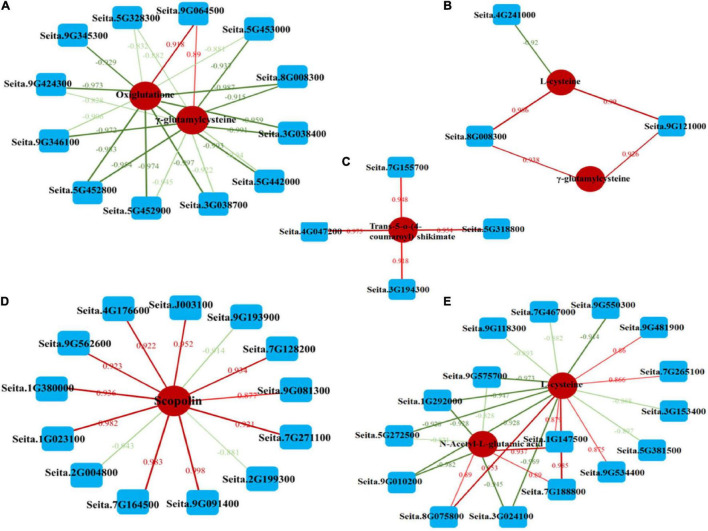
Coexpression analysis of DEGs and DEMs based on Pearson’s correlation. **(A,B)** Interaction network of DEGs and DEMs involved in glutathione biosynthesis (ko00480) in GCK vs. GT and LCK vs. LT. **(C,D)** Interaction network of DEGs and DEMs involved in phenylpropanoid biosynthesis (ko00940) in GCK vs. GT and LCK vs. LT. **(E)** Interaction network of DEGs and DEMs involved in amino acid biosynthesis (ko01230) in LCK vs. LT. Lines colored in “red” and “green” display positive and negative correlation, respectively, according to Pearson’s correlation coefficient of >0.8 or <–0.8, respectively.

To further examine the relationship between DEGs and DEMs in millet under atrazine stress, a coexpression network analysis was conducted. Oxiglutatione, L-cysteine, and γ-glutamylcysteine were found to be related to glutathione metabolism (ko00480, Supplementary Figure 3 and Figures 4A,B) and more highly regulated in GCK vs. GT than in LCK vs. LT (Table 6). By contrast, except for glutathione synthesis, *trans-*5-o-(4-coumaroyl)shikimate and scopolin were highly enriched in LCK vs. LT and were correlated with phenylpropanoid biosynthesis (ko00940, Supplementary Figure 3 and Figures 4C,D). Moreover, the coexpression network of DEGs and DEMs in GCK vs. GT was mainly enriched in the biosynthesis of amino acids (ko01230, Supplementary Figure 3 and Figure 4E), such as L-cysteine, and N-acetyl-L-glutamic acid was more highly regulated in GCK vs. GT than in LCK vs. LT (Table 6).

**TABLE 6:** Regulation of metabolites in the two samples under atrazine stress.

No.	Meta ID	Meta name	GCK vs. GT	LCK vs. LT
1	pmb3264	Oxiglutatione	Up	Unchanged
2	pme2563	γ-glutamylcysteine	Up	Down
3	pmb0751	*Trans-*5-o-(4-coumaroyl)shikimate	Down	Up
4	mws1077	Scopolin	Down	Up
5	pme0195	L-cysteine	Up	Down
6	pme0075	N-Acetyl-L-glutamic acid	Up	Down
7	pme0006	L-Proline	Up	Down

### Integrated Analysis of Genes and Metabolites Involved in Glutathione Biosynthesis in Millet Under Atrazine Stress

**FIGURE 5:**
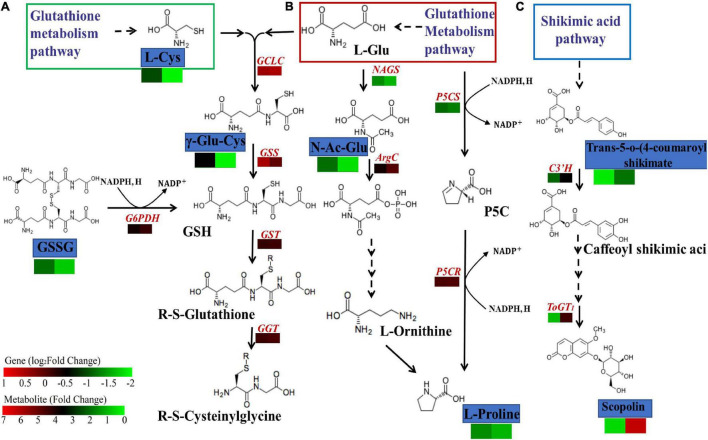
DEGs and DEMs involved in glutathione biosynthesis **(A)**, amino acid biosynthesis **(B)**, and shikimic acid **(C)** metabolism pathway in response to atrazine stress. The rectangles are divided into two equal parts, the left part represents DEGs or DEMs in GA2, whereas the right part represents DEGs or DEMs in LG31. The colors in the rectangles represent the genes or metabolites that were regulated under atrazine stress (red indicates upregulation; black indicates no changes; and green indicates downregulation). *GCLC*, glutamate cysteine ligase catalytic subunit; *GSS*, glutathione synthase; *GST*, glutathione S-transferase; *GGT*, γ-glutamyltranspeptidase; *G6PDH*, glucose-6-phosphate 1-dehydrogenase; *ToGT1*, scopoletin glucosyltransferase; *C3*′*H*, 5-o-(4-coumaroyl)-D-quinate 3′-monooxygenase; *NAGS2*, N-acetyl-L-glutamate synthetase; *ArgC*, N-acetyl-gamma-glutamyl-phosphate reductase; *P5CS*, delta-1-pyrroline-5-carboxylate synthetase; *P5CR*, pyrroline-5-carboxylate reductase.

To further analyze the effects of atrazine on genes and metabolites involved in glutathione biosynthesis in millet, the networks of DEGs and DEMs involved in glutathione metabolism pathway (ko00480) were analyzed (Figure 5A and Supplementary Table 8). The results showed that 12 and 4 genes were differentially expressed in GA2 and LG31 at transcriptional levels, respectively. Compared with the CK group, the expression of all genes, except Seita.9G06450 and Seita.4G241000, was downregulated in the treatment group. The functions of all these genes were related to glutathione S-transferase (EC: 2.5.1.18). In addition, other genes in this pathway, such as *GCLC* (encoding for glutamate cysteine ligase catalytic subunit, Seita.3G050000), *GGT* (encoding for γ-glutamyl transpeptidase, Seita.5G104100), and *G6PDH* (encoding for glucose-6-phosphate 1-dehydrogenase, Seita.9G424300), were downregulated, whereas *GSS* (encoding for glutathione synthase, Seita.3G348900) was upregulated in GA2 (Supplementary Table 9). DEMs such as oxiglutatione, L-cysteine, and γ-glutamylcysteine were related to glutathione metabolism and more highly regulated in GCK vs. GT than in LCK vs. LT. The results showed that the DEGs and DEMs related to glutathione metabolism in GA2 were involved in the response to atrazine stress.

### Integrated Analysis of Genes and Metabolites Involved in Amino Acid Biosynthesis in Millet Under Atrazine Stress

To investigate the effect of atrazine stress on the expressions of genes and metabolites involved in amino acid biosynthesis, the interaction of DEGs and DEMs involved in amino acid biosynthesis (ko01230) was analyzed (Figure 5B and Supplementary Table 10). We found that the expression of the DEMs N-acetyl-L-glutamic acid and L-proline was more highly regulated in GCK vs. GT than in LCK vs. LT. However, almost all DEGs related to this pathway were downregulated in GA2 (Supplementary Table 9), including the genes that may be regulated by enzymes related to the two abovementioned metabolites, such as *NAGS* (encoding for N-acetyl-L-glutamate synthetase, Seita.7G188800) and *P5CS* (encoding for delta-1-pyrroline-5-carboxylate synthetase, Seita.3G220500), except for *ArgC* (encoding for N-acetyl-gamma-glutamyl-phosphate reductase, Seita.9G167800). The results showed that the DEGs and DEMs related to amino acid biosynthesis in GA2 were involved in the response to atrazine stress.

### Integrated Analysis of Genes and Metabolites Involved in Phenylpropanoid Biosynthesis in Millet Under Atrazine Stress

To examine the effects of atrazine stress on the expression of genes and metabolites involved in phenylpropanoid biosynthesis, the interaction of DEGs and DEMs involved in amino acid biosynthesis was analyzed (Figure 5C and Supplementary Table 10). DEGs such as *ToGT1* (encoding for scopoletin glucosyltransferase, Seita.5G239500) and *C3*′*H* [encoding for 5-o-(4-coumaroyl)-D-quinate 3′-monooxygenase, Seita.3G194300] were upregulated in LG31 (Supplementary Table 9). Moreover, the DEMs *trans-*5-o-(4-coumaroyl)shikimate and scopolin were highly enriched in LCK vs. LT and were correlated to phenylpropanoid biosynthesis (ko00940). The results showed that the DEGs and DEMs related to phenylpropanoid biosynthesis in LG31 were jointly involved in the response to atrazine stress.

### Real-Time Quantitative Reverse Transcription PCR Validation of Differentially Expressed Genes in Millet Under Atrazine Stress

To validate the accuracy of the RNA-Seq data, the expression levels of approximately 34 DEGs (Supplementary Table 11) that were related to glutathione, phenylpropanoid, and amino acid biosyntheses, such as *GSTU6*, *HST*, *GLN1-3*, and GSTF1, were measured using qRT-PCR. The results were similar to those of RNA-Seq (log_2_FC), which verified the reproducibility and credibility of the RNA-Seq data.

## Discussion

In recent years, the rapid increase in the use of the herbicide atrazine in field-planted maize has resulted in a serious impact on the next rotation crop and environment. Considering that millet is a suitable rotation crop for maize, it is important to screen and obtain atrazine-tolerant varieties of millet for maize rotation. In this study, we analyzed the effects of atrazine stress on glutathione metabolism and amino acid and phenylpropanoid biosyntheses by performing an integrated analysis of the transcriptome and metabolome. The results showed that millet plants can be damaged by atrazine stress.

### Glutathione Biosynthesis Was Enhanced in GA2 Millet Variety Under Atrazine Stress

several studies indicated that atrazine stress can lead to excessive accumulation of ROS in plant cells, resulting in the peroxidation of membrane lipids and oxidative damage to the cell membrane, proteins, and DNA (Bai et al., 2015; Baxter et al., 2015; Erinle et al., 2018; Singh et al., 2018). However, to avoid damage caused by ROS, plants have evolved a complex detoxification system (Yuan et al., 2007). For example, plants can use ROS scavenging enzymes (Superoxide Dismutase, Peroxidases, and Catalase) and non-enzymatic antioxidants (glutathione, glutathione reductase, and glutathione-Px) to scavenge excess ROS (Sharif et al., 2018). If the excess ROS is not eliminated by detoxification system, which could be marked by the accumulation of MDA (Bai et al., 2015). Sathish et al. (2022) found MDA content in *Zea mays* L. was significantly increased by water deficit stress. In the present study, the MDA content in GA2 was lower than that in LG31 under atrazine stress, indicating that the degree of peroxidation damage caused by atrazine in GA2 was less to maintain relatively better membrane stability. Moreover, the activities of ROS scavenging enzymes were not well detected in this study. These results indicate that ROS scavenging in millet plants mainly occurs by increasing the levels of non-enzymatic antioxidants, which corroborates the findings of a previous study on fulvic acid used for alleviating drought stress in tea plants (Sun J. H. et al., 2020).

Glutathione is a thiol tripeptide with a low molecular weight that widely exists in all kinds of cells and functions as a non-enzymatic antioxidant that scavenges for H_2_O_2_, ^1^O_2_, OH^•^, and ${\text{O}}_{2}^{•-}$ and protects different biomolecules to maintain ROS homeostasis (Das and Roychoudhury, 2014). Glutathione S-transferases (GSTs) can convert various herbicides using glutathione into non-toxic and water-soluble forms, making the plant resistant and reducing the damage to crops caused by herbicides (Shimabukuro et al., 1970). In this study, glutathione levels were not significantly different in the atrazine-resistant GA2 and atrazine-sensitive LG31 variety. Oxidized glutathione (GSSG) levels were increased in GA2, but showed no change in LG31. These results imply that the dynamic equilibrium between glutathione and GSSG may fluctuate because glutathione participates in resistance to environmental stress in GA2. Moreover, a previous study reported that maize and grain sorghum, as crops naturally tolerant to atrazine, showed rapid detoxification resulting from the high constitutive activity of GSTs (Timmerman, 1989). Similarly, studies on *Lolium rigidum* in Australia and *Alopecurus myosuroides* in the United Kingdom have also proved the detoxification mechanisms of GST (Cummins et al., 2013; Yu and Powles, 2014). In the present study, nine genes with functions related to GST were detected in GA2, but the expressions of the GST genes in the atrazine treatment group were downregulated at the transcriptional level. The reason for this may be that gene transcription occurs before substance metabolism. Therefore, we deduced that the glutathione pathway plays an important role in the resistance of GA2 to atrazine stress.

### Proline Levels Increased in the GA2 Millet Variety Under Atrazine Stress

Proline, an osmotic regulator, is the main amino acid for the stabilization and protection of cell membranes and proteins under certain stress conditions, such as drought, salinity, and low and high temperatures (Rivero et al., 2014; Hasanuzzaman et al., 2019). The biosynthesis of proline mainly depends on glutamate, and the rate-limiting step in this pathway is catalyzed by pyrroline-5-carboxylate synthetase (P5CS; Rejeb et al., 2014). Previous studies reported that *P5CS1* expression can be induced by drought, salt, abscisic acid (ABA), light, nitric oxide, and pathogens to improve the concentration of proline in Arabidopsis (Savouré et al., 1995; Strizhov et al., 1997; Hayashi et al., 2000; Fabro et al., 2004; Zhao et al., 2009). In general, tolerant genotypes of crop plants accumulated more osmoprotectants with high concentrations than sensitive genotypes under stress. For example, a near-isogenic line of spring barley genotypes (NIL143) that contains the wild allele *pyrroline-5-carboxylate synthase 1-P5cs1* was observed higher proline concentrations in leaf and root and less severe symptoms of drought compared with any other genotypes under reduced water availability conditions (Frimpong et al., 2021).

In our study, we found the DEGs related to the proline biosynthesis pathway were all downregulated, including *P5CS* (Seita.3G220500). While proline was significantly accumulated in the GA2 group under atrazine stress. Many researchers also suggested that proline can not only improve osmotic tolerance in plants cell but also reduce ROS generation and scavenge excessive free ROS (Smirnoff and Cumbes, 1989; Alia et al., 1991; Shalata and Neumann, 2001). Recent studies also showed a positive correlation between proline and ROS levels under abiotic stress or exogenous treatment with H_2_O_2_ (Yang et al., 2009; Lopez-Delacalle et al., 2020). Previous studies showed that many metabolic mechanisms are involved in the response of plants to abiotic stress, including the production of signal molecules, activation and inactivation of gene expression, and synthesis of functional proteins (Zushi et al., 2014; Zandalinas et al., 2020). Among these, gene expression is rapid and occurs before protein synthesis. Thus, we only detected significantly increased levels of proline in the atrazine-tolerant GA2 variety, suggesting that proline may help in scavenging excessive ROS produced by atrazine stress and protecting cells from damage.

### Scopoletin Levels Increased in the LG31 Millet Variety Under Atrazine Stress

To protect plant themselves from various environmental stress factors, plant cells have developed an inducible defense mechanism that involves the production of toxic chemicals or antimicrobial compounds, such as secondary metabolites (Kliebenstein and Osbourn, 2012; Tiku, 2018). Scopoletin and its glucoside scopolin are coumarins, which are one of the secondary metabolite classes (Kai et al., 2006). The biosynthesis of scopoletin mostly occurs via the shikimic acid pathway (Bayoumi et al., 2008). Previous studies reported that treatment with CuCl_2_, shortwave UV, and Triton X-100 induces scopoletin accumulation in sunflower leaves (Gutierrez et al., 1995). Scopoletin is also produced in leaves in response to pathogen attack (Barón et al., 2016). Moreover, its role in abiotic stress may be as an antioxidant for scavenging ROS to reduce oxidative stress in plant cells (Chong et al., 2002; Döll et al., 2018). In this study, DEMs and DEGs in the synthesis pathways of scopolin and metabolites [scopolin and *trans-*5-o-(4-coumaroyl)shikimate] as well as genes (*ToGT1* and *C3′H*) were significantly upregulated in LG31 and downregulated in GA2. These results showed that scopolin may be accumulated in LG31 as an antioxidant to scavenge excessive ROS produced by atrazine stress.

## Conclusion

In summary, we systematically analyzed the effects of the mechanisms of atrazine stress on millet plants by using metabolomics and transcriptomics approaches. Our results showed that many biological metabolic pathways are affected by atrazine stress in millet leaves, such as glutathione metabolism, amino acid biosynthesis, and phenylpropanoid biosynthesis. Meanwhile, our results also revealed that the GA2 variety enhanced atrazine tolerance probably by changing glutathione metabolism and amino acid biosynthesis. Moreover, the accumulation of scopolin in LG31 may play an important role in the response to atrazine stress.

## Data Availability Statement

The datasets presented in this study can be found in online repositories. The names of the repository/repositories and accession number(s) can be found below: National Center for Biotechnology Information (NCBI) BioProject database under accession number PRJNA751769.

## Author Contributions

XM and SG were responsible for the conceived and designed the experiments. WS was responsible for designed the experiments and revised the manuscript. LS was responsible for the data analysis, literature search, and manuscript preparation. LL, YW, and WY performed the experiments and data analysis. YF and DW contributed to reagents, materials, and analysis tools. All authors read and approved the final version of the manuscript.

## Conflict of Interest

WS was employed by Heilongjiang HYHC Company. The remaining authors declare that the research was conducted in the absence of any commercial or financial relationships that could be construed as a potential conflict of interest.

## Publisher’s Note

All claims expressed in this article are solely those of the authors and do not necessarily represent those of their affiliated organizations, or those of the publisher, the editors and the reviewers. Any product that may be evaluated in this article, or claim that may be made by its manufacturer, is not guaranteed or endorsed by the publisher.

## Footnotes

1
www.biomarker.com.cn

2
https://phytozome.jgi.doe.gov/pz/portal.html#!info?alias=Org_Sitalica

3
http://metlin.scripps.edu
