# Supplementary figures and images for: Integration of Metabolomics and Transcriptomics for Investigating the Tolerance of Foxtail Millet (Setaria italica) to Atrazine Stress
Source: Front Plant Sci. 2022 Jun 10;13:890550. doi: 10.3389/fpls.2022.890550 (PMC9226717; doi:10.3389/fpls.2022.890550)

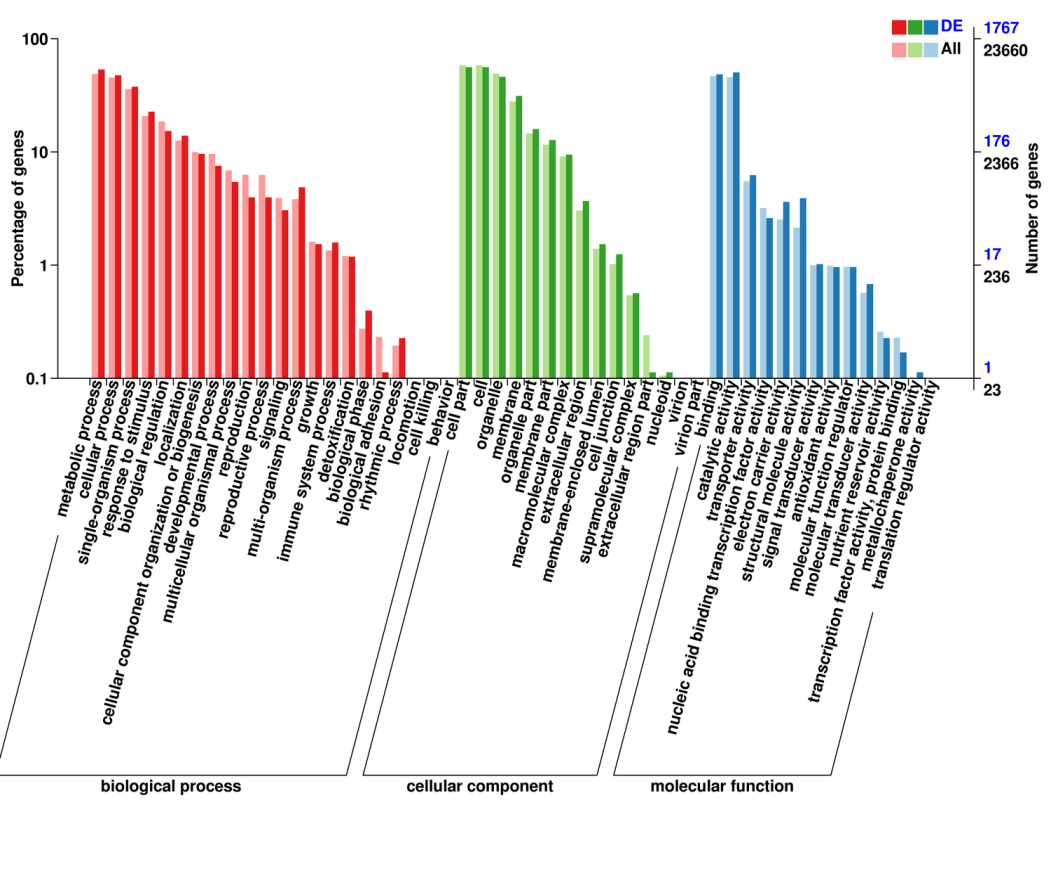

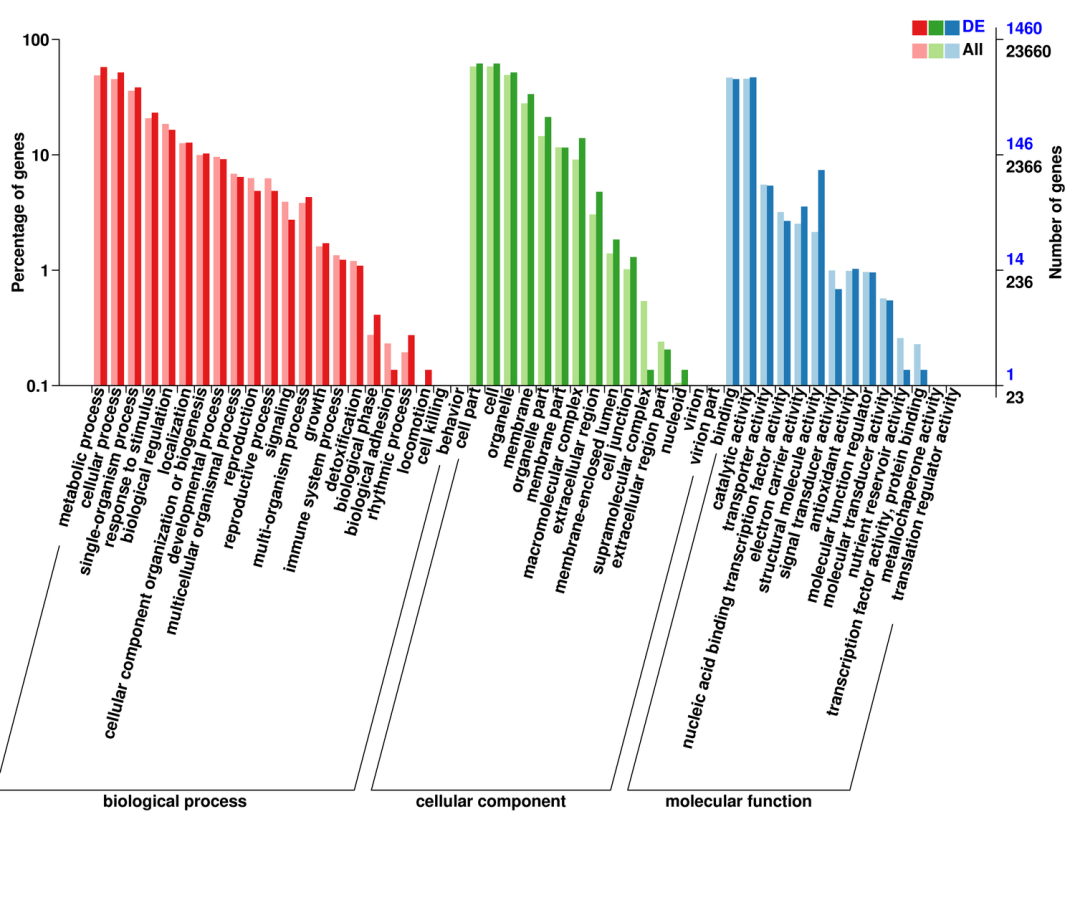


**B**

**A**

Supplemental Fig. 1. The GO analysis of DEGs

Note: A, GCK&GT; B, LCK&LT

Supplement: Supplementary file 1 [file Table_1.DOCX]

## Slide 1
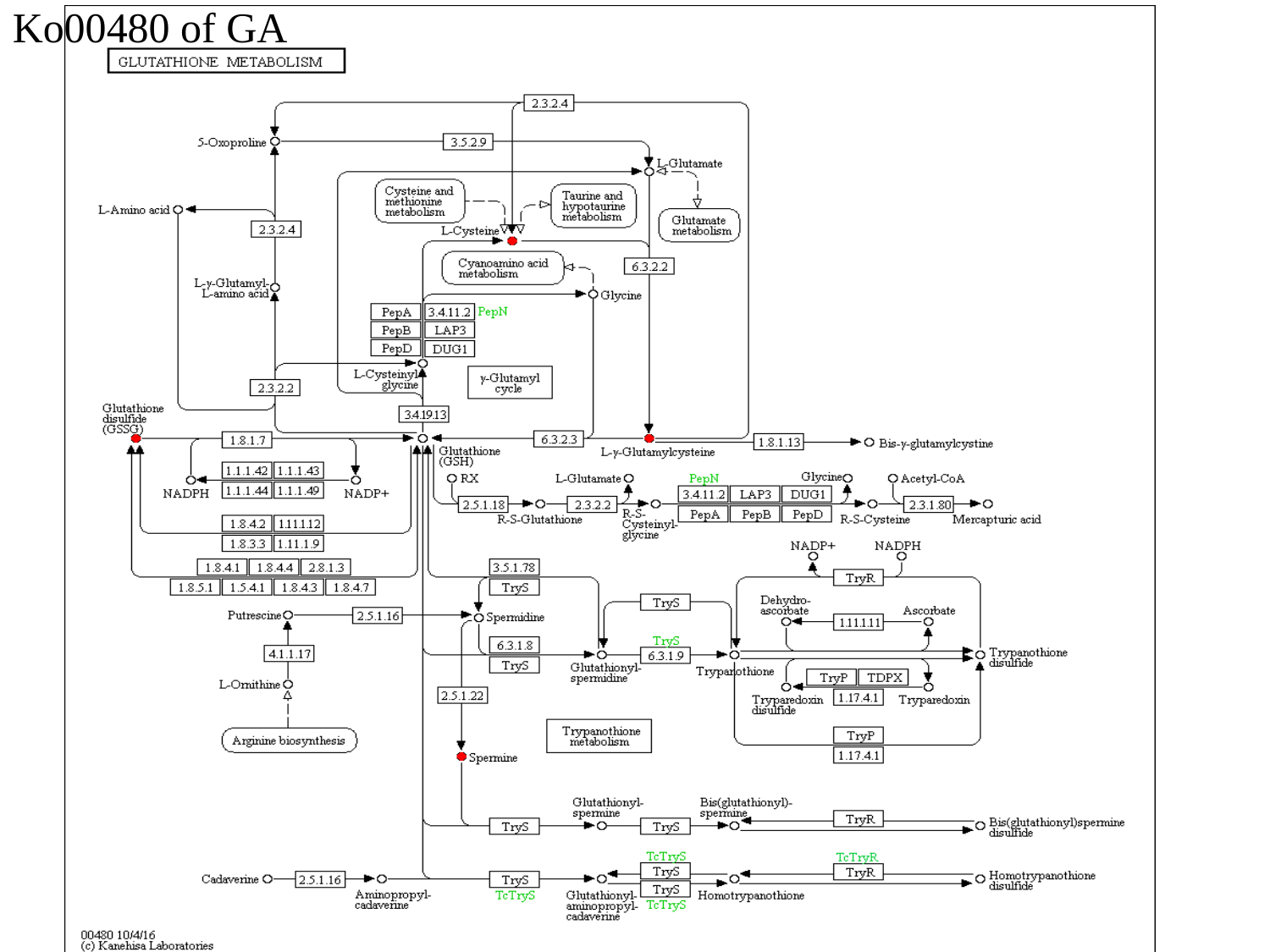

Ko00480 of GA

## Slide 2
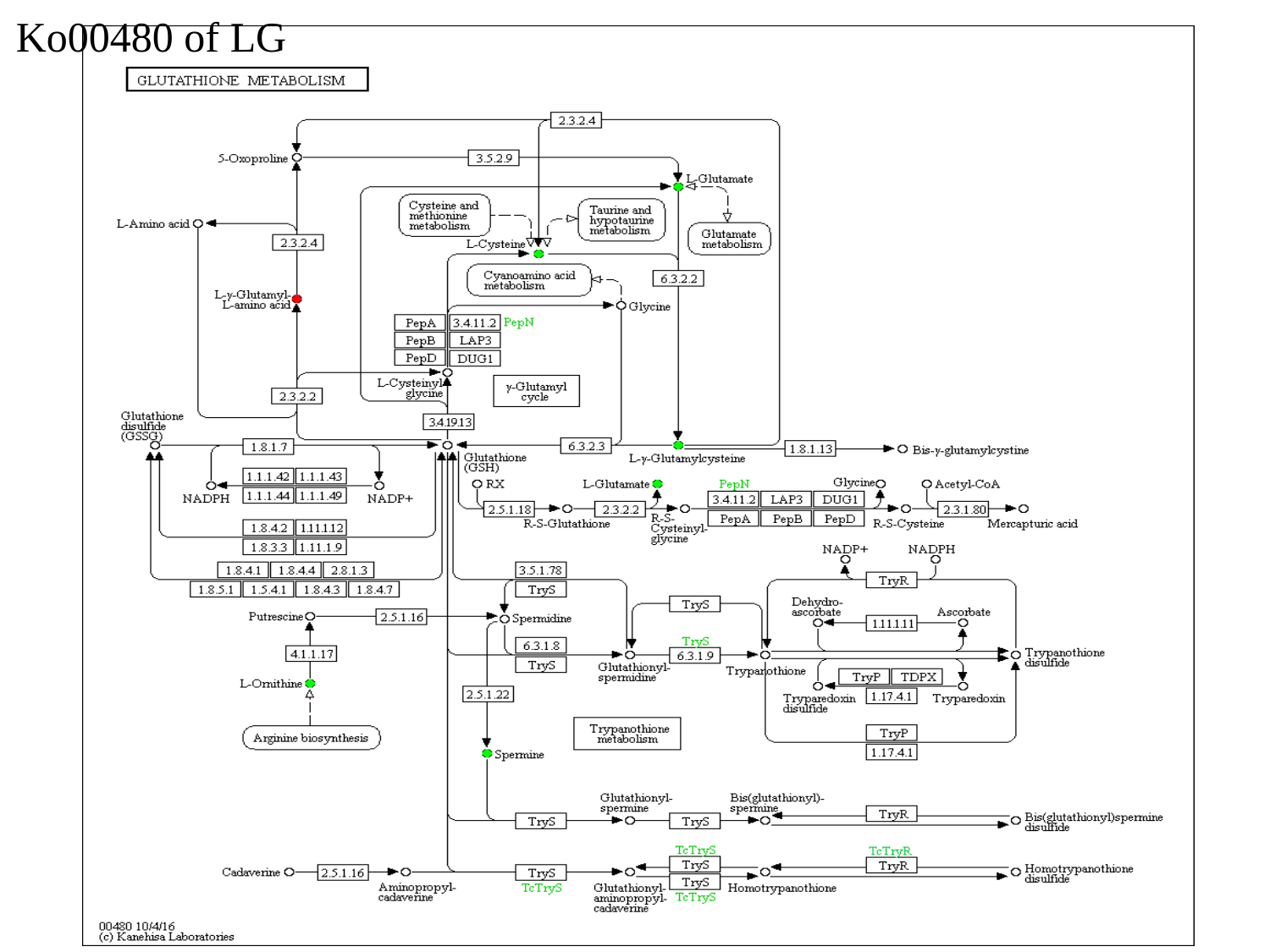

Ko00480 of LG

## Slide 3
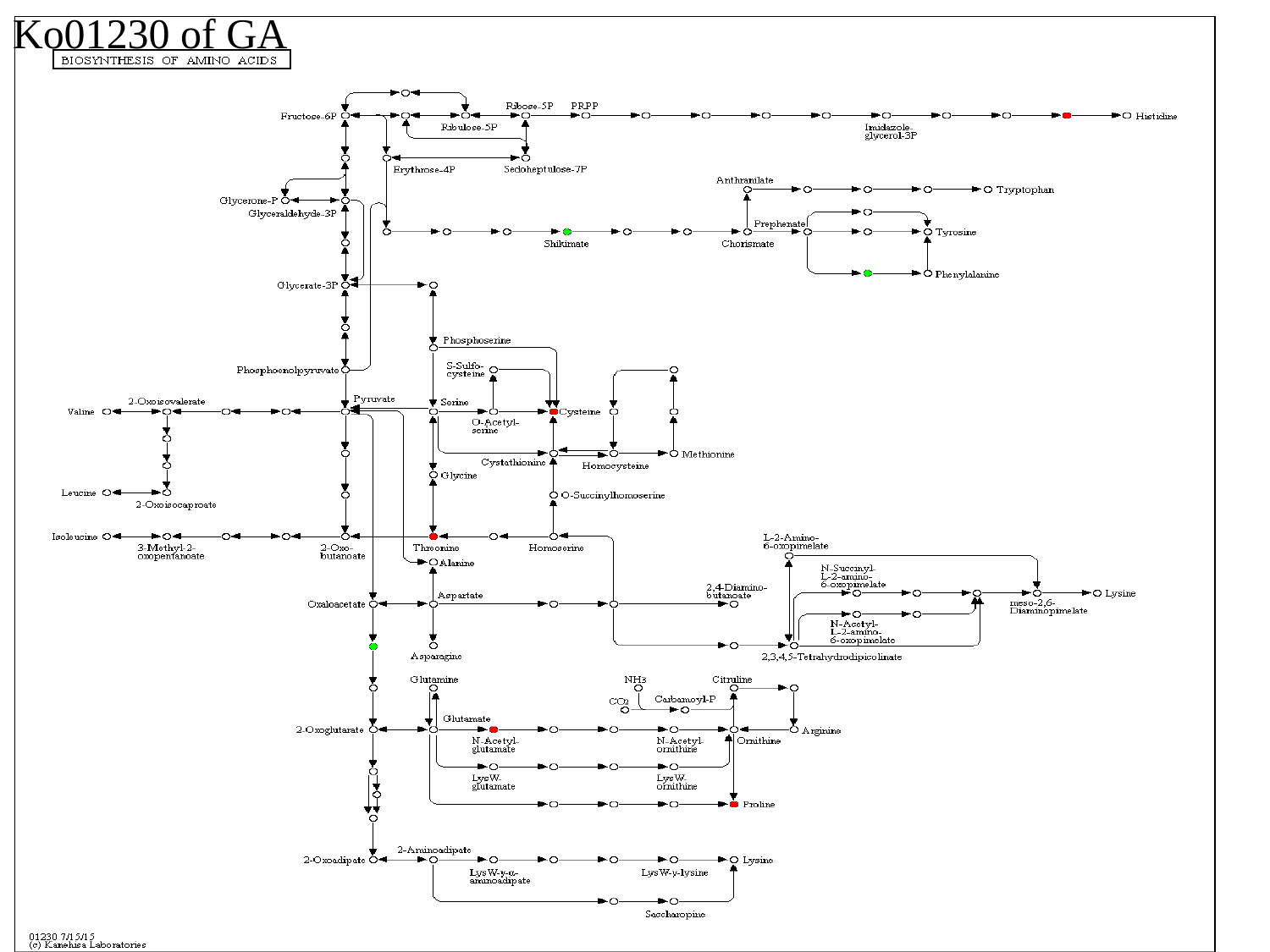

Ko01230 of GA

## Slide 4
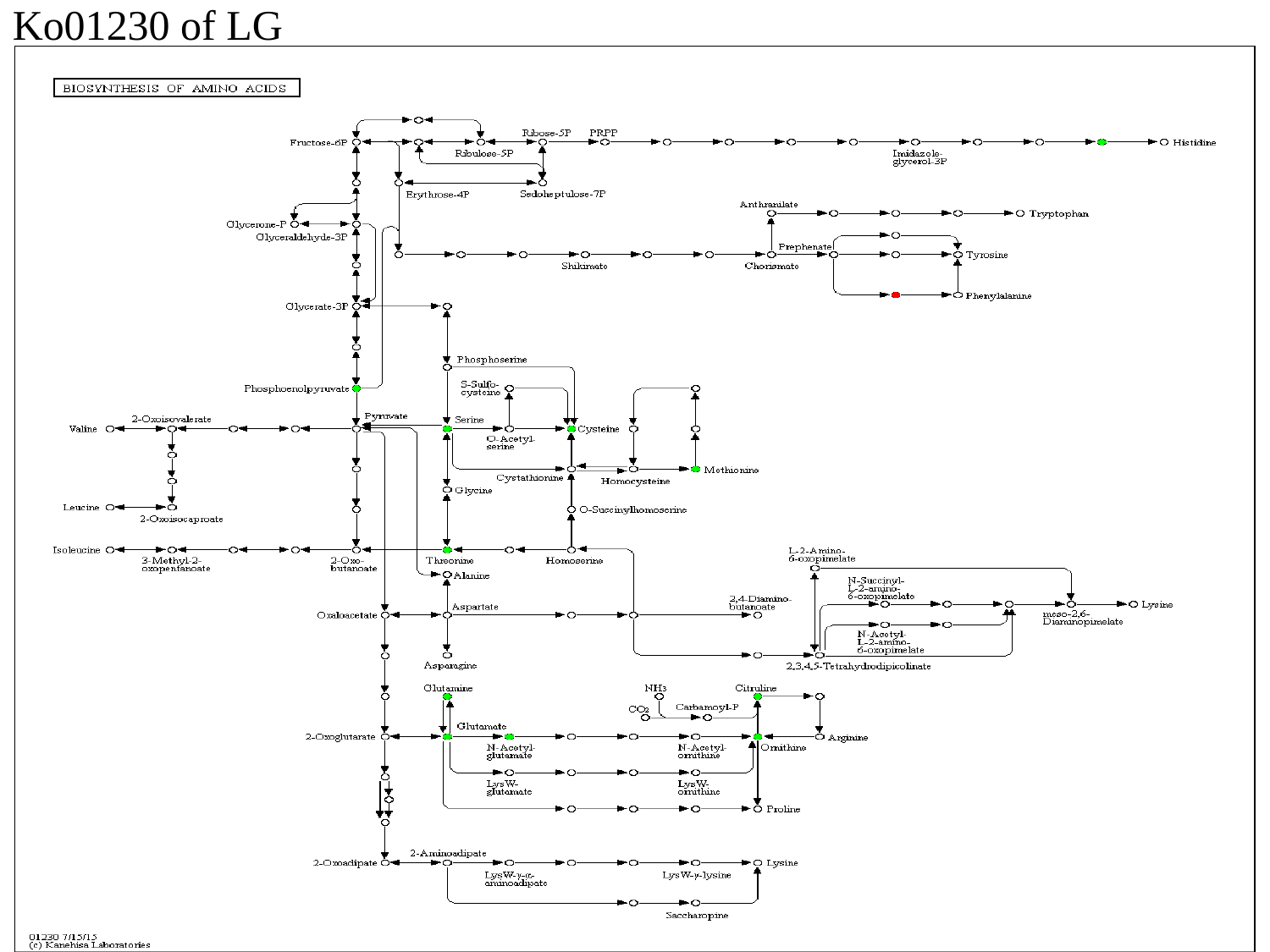

Ko01230 of LG

## Slide 5
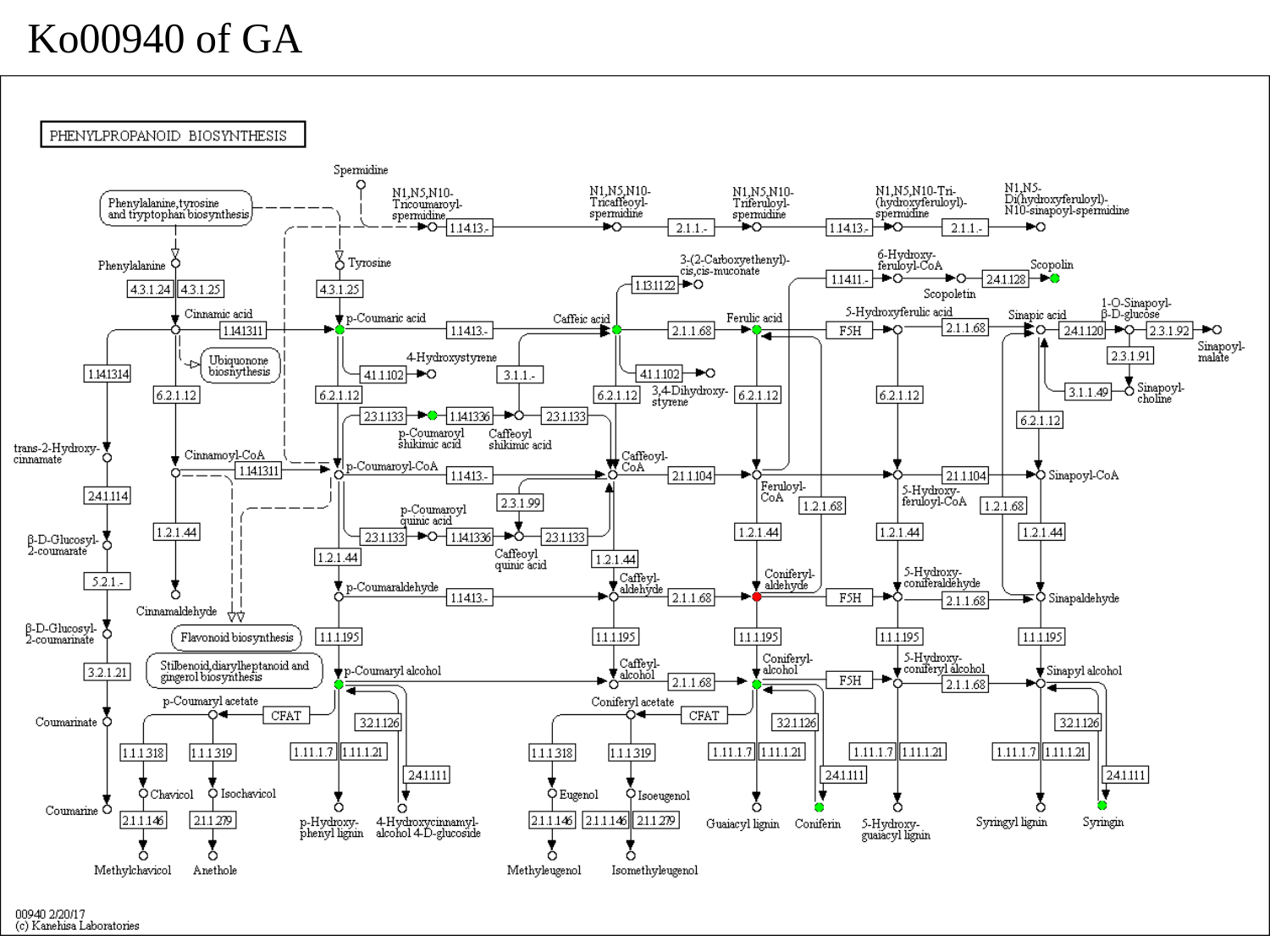

Ko00940 of GA

## Slide 6
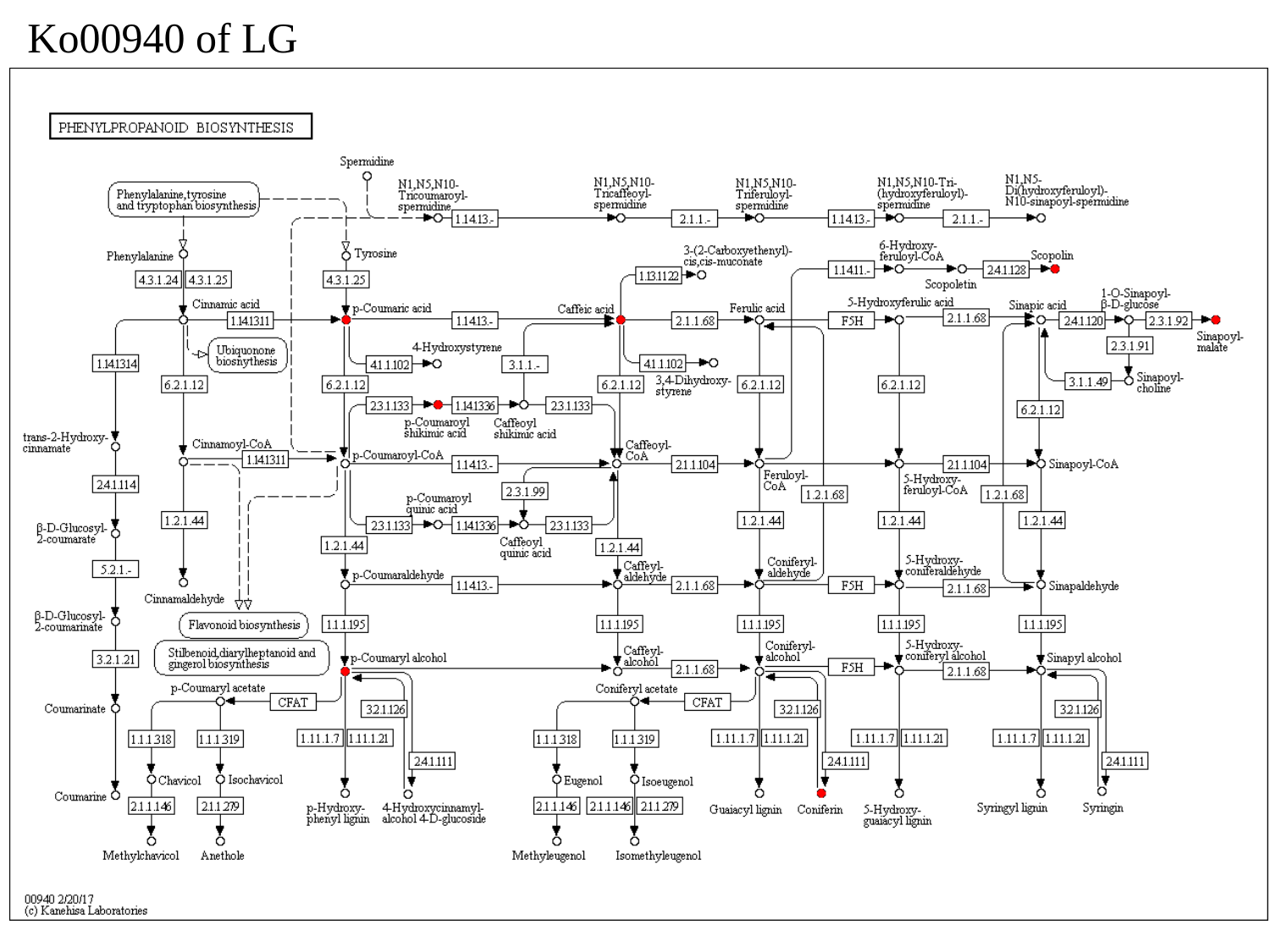

Ko00940 of LG

Supplement: Supplementary file 3 [file Presentation_1.PPTX]
