# Supplementary material for: Integration of Metabolomics and Transcriptomics for Investigating the Tolerance of Foxtail Millet (Setaria italica) to Atrazine Stress
Source: Front Plant Sci. 2022 Jun 10;13:890550. doi: 10.3389/fpls.2022.890550 (PMC9226717; doi:10.3389/fpls.2022.890550)
Supplement: Supplementary file 2 [file Table_2.DOCX]

**
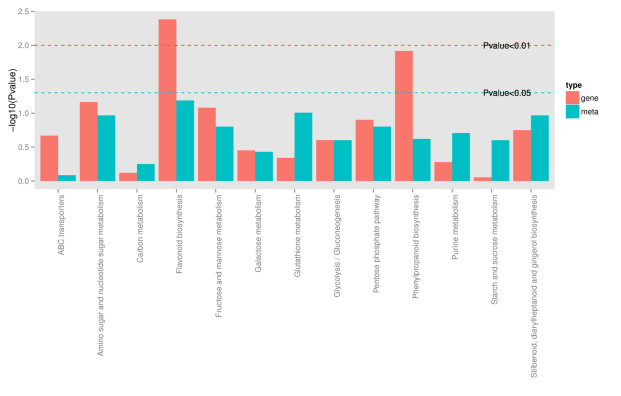
**

A

**
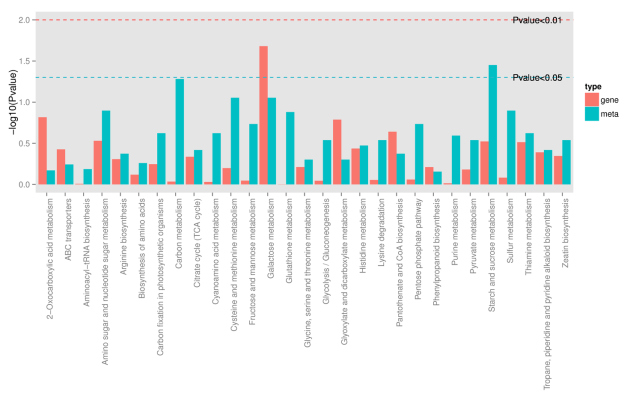
**

B

**Supplemental Fig. 2.** The KEGG analysis between DEMs and DEGs in resistant/sensitive millet leaves under atrazine stress. A. GCKvsGT; B. LCKvsLT
