# Supplementary material for: Integration of Metabolomics and Transcriptomics for Investigating the Tolerance of Foxtail Millet (Setaria italica) to Atrazine Stress
Source: Front Plant Sci. 2022 Jun 10;13:890550. doi: 10.3389/fpls.2022.890550 (PMC9226717; doi:10.3389/fpls.2022.890550)
Supplement: Supplementary file 11 [file Table_10.DOCX]

**The expression genes of ko00480 in GCKvsGT**

| **NO.** | **Gene ID** | **RefSeq** | **Expression** | **E value** |
| --- | --- | --- | --- | --- |
|  | Seita.3G038400 | glutathione S-transferase GSTF1 | down | 5.44942e-160 |
|  | Seita.3G038700 | glutathione S-transferase 4-like | down | 2.19862e-158 |
|  | Seita.5G328300 | glutathione S-transferase 1 | down | 1.77581e-156 |
|  | Seita.5G442000 | glutathione S-transferase 3 | down | 6.39548e-157 |
|  | Seita.5G452800 | glutathione transferase GST 23-like | down | 3.01171e-157 |
|  | Seita.5G452900 | glutathione S-transferase | down | 1.22256e-168 |
|  | Seita.5G453000 | glutathione S-transferase | down | 1.25249e-152 |
|  | Seita.8G008300 | glutathione transferase GST 23-like | down | 1.21003e-166 |
|  | Seita.9G064500 | glutathione S-transferase GSTU1 | up | 1.44639e-119 |
|  | Seita.9G345300 | glutathione S-transferase GSTU6 | down | 4.74526e-135 |
|  | Seita.9G346100 | glutathione S-transferase GSTU6 | down | 1.80321e-167 |
|  | Seita.9G424300 | glucose-6-phosphate 1-dehydrogenase | down | 0 |

**The expression genes of ko00480 in LCKvsLT**

| **NO.** | **Gene ID** | **RefSeq** | **Expression** | **E value** |
| --- | --- | --- | --- | --- |
| 1 | Seita.8G008300 | glutathione transferase GST 23-like | down | 1.21003e-166 |
| 2 | Seita.4G241000 | glutathione transferase GST 23-like | up | 1.20767e-175 |
| 3 | Seita.9G121000 | microsomal glutathione S-transferase 3-like | down | 3.50838e-96 |
| 4 | Seita.9G345300 | glutathione S-transferase GSTU6 | down | 4.74526e-135 |
