# Supplementary material for: Integration of Metabolomics and Transcriptomics for Investigating the Tolerance of Foxtail Millet (Setaria italica) to Atrazine Stress
Source: Front Plant Sci. 2022 Jun 10;13:890550. doi: 10.3389/fpls.2022.890550 (PMC9226717; doi:10.3389/fpls.2022.890550)
Supplement: Supplementary file 13 [file Table_12.DOCX]

**The expression genes of ko01230 in GCKvsGT**

| **NO.** | **Gene ID** | **RefSeq** | **Expression** | **E value** |
| --- | --- | --- | --- | --- |
|  | Seita.1G147500 | 4-hydroxy-tetrahydrodipicolinate reductase [EC:1.17.1.8] | down | 0 |
|  | Seita.1G160800 | fructose-bisphosphate aldolase, class I [EC:4.1.2.13] | down | 0 |
|  | Seita.2G151900 | indole-3-glycerol phosphate synthase [EC:4.1.1.48] | down | 0 |
|  | Seita.2G205000 | 6-phosphofructokinase 1 [EC:2.7.1.11] | down | 0 |
|  | Seita.2G394800 | 3-deoxy-7-phosphoheptulonate synthase [EC:2.5.1.54] | down | 0 |
|  | Seita.3G111200 | 6-phosphofructokinase 1 [EC:2.7.1.11] | down | 0 |
|  | Seita.3G153400 | glutamate synthase (NADPH/NADH) [EC:1.4.1.13 1.4.1.14] | up | 0 |
|  | Seita.3G220500 | delta-1-pyrroline-5-carboxylate synthetase [EC:2.7.2.11 1.2.1.41] | down | 0 |
|  | Seita.3G306100 | enolase [EC:4.2.1.11] | down | 0 |
|  | Seita.5G419900 | fructose-bisphosphate aldolase [EC:4.1.2.13] | down | 0 |
|  | Seita.6G117900 | bifunctional aspartokinase / homoserine dehydrogenase 1 [EC:2.7.2.4 1.1.1.3] | down | 0 |
|  | Seita.6G167400 | D-3-phosphoglycerate dehydrogenase 3 [EC:1.1.1.95] | down | 0 |
|  | Seita.6G250100 | dihydroxy-acid dehydratase [EC:4.2.1.9] | down | 0 |
|  | Seita.7G087700 | arogenate/prephenate dehydratase [EC:4.2.1.91 4.2.1.51] | down | 0 |
|  | Seita.7G188800 | acetylglutamate kinase-like [EC:2.7.2.8] | down | 0 |
|  | Seita.7G265100 | D-3-phosphoglycerate dehydrogenase [EC:1.1.1.95] | down | 0 |
|  | Seita.7G315100 | phosphoglycerate mutase-like [EC:5.4.2.12] | down | 4.79092e-134 |
|  | Seita.7G315200 | phosphoglycerate mutase-like [EC:5.4.2.12] | down | 2.59193e-87 |
|  | Seita.8G050800 | fructose-bisphosphate aldolase [EC:4.1.2.13] | up | 0 |
|  | Seita.8G075800 | acetolactate synthase I/III small subunit [EC:2.2.1.6] | down | 0 |
|  | Seita.9G010200 | aspartate kinase [EC:2.7.2.4] | up | 0 |
|  | Seita.9G379300 | 3-deoxy-7-phosphoheptulonate synthase [EC:2.5.1.54] | down | 0 |
|  | Seita.9G481900 | branched-chain amino acid aminotransferase [EC:2.6.1.42] | down | 0 |
|  | Seita.9G506400 | LL-diaminopimelate aminotransferase [EC:2.6.1.83] | down | 0 |
|  | Seita.9G534400 | phosphoserine aminotransferase [EC:2.6.1.52] | down | 0 |

**The expression genes of ko01230 in LCKvsLT**

| **NO.** | **Gene ID** | **RefSeq** | **Expression** | **E value** |
| --- | --- | --- | --- | --- |
|  | Seita.9G575700 | branched-chain amino acid aminotransferase [EC:2.6.1.42] | up | 0 |
|  | Seita.9G550300 | serine O-acetyltransferase [EC:2.3.1.30] | up | 0 |
|  | Seita.9G534400 | phosphoserine aminotransferase [EC:2.6.1.52] | down | 0 |
|  | Seita.9G481900 | branched-chain amino acid aminotransferase [EC:2.6.1.42] | down | 0 |
|  | Seita.9G416100 | 2,3-bisphosphoglycerate-independent phosphoglycerate mutase [EC:5.4.2.12] | down | 0 |
|  | Seita.9G118300 | glutamine synthetase [EC:6.3.1.2] | up | 0 |
|  | Seita.9G010200 | aspartate kinase [EC:2.7.2.4] | up | 0 |
|  | Seita.8G075800 | acetolactate synthase I/III small subunit [EC:2.2.1.6] | down | 0 |
|  | Seita.7G265100 | D-3-phosphoglycerate dehydrogenase [EC:1.1.1.95] | down | 0 |
|  | Seita.7G188800 | acetylglutamate kinase [EC:2.7.2.8] | down | 0 |
|  | Seita.7G046700 | ribose 5-phosphate isomerase A [EC:5.3.1.6] | up | 8.01288e-180 |
|  | Seita.6G250100 | dihydroxy-acid dehydratase [EC:4.2.1.9] | down | 0 |
|  | Seita.6G167400 | D-3-phosphoglycerate dehydrogenase [EC:1.1.1.95] | down | 0 |
|  | Seita.5G381500 | transketolase [EC:2.2.1.1] | up | 0 |
|  | Seita.5G272500 | glutamate synthase (NADPH/NADH) [EC:1.4.1.13 1.4.1.14] | up | 0 |
|  | Seita.3G153400 | glutamate synthase (NADPH/NADH) [EC:1.4.1.13 1.4.1.14] | up | 0 |
|  | Seita.3G024100 | glutamine synthetase [EC:6.3.1.2] | up | 0 |
|  | Seita.1G292000 | histidinol-phosphate aminotransferase [EC:2.6.1.9] | up | 0 |
|  | Seita.1G147500 | 4-hydroxy-tetrahydrodipicolinate reductase [EC:1.17.1.8] | down | 0 |

**The expression genes of ko00940 in GCKvsGT**

| **NO.** | **Gene ID** | **RefSeq** | **Expression** | **E value** |
| --- | --- | --- | --- | --- |
|  | Seita.1G065800 | 4-coumarate--CoA ligase [EC:6.2.1.12] | down | 0 |
|  | Seita.1G240200 | phenylalanine/tyrosine ammonia-lyase [EC:4.3.1.25] | down | 0 |
|  | Seita.1G240300 | phenylalanine ammonia-lyase [EC:4.3.1.24] | down | 0 |
|  | Seita.1G240500 | phenylalanine ammonia-lyase [EC:4.3.1.24] | down | 0 |
|  | Seita.1G380000 | peroxidase [EC:1.11.1.7] | up | 0 |
|  | Seita.2G004800 | peroxidase [EC:1.11.1.7] | down | 0 |
|  | Seita.2G199300 | cinnamyl-alcohol dehydrogenase [EC:1.1.1.195] | down | 0 |
|  | Seita.2G254100 | beta-glucosidase [EC:3.2.1.21] | down | 0 |
|  | Seita.2G431200 | peroxidase [EC:1.11.1.7] | down | 0 |
|  | Seita.2G431300 | peroxidase [EC:1.11.1.7] | down | 0 |
|  | Seita.3G052400 | peroxidase [EC:1.11.1.7] | down | 0 |
|  | Seita.3G194300 | coumaroylquinate(coumaroylshikimate) 3'-monooxygenase [EC:1.14.13.36] | down | 0 |
|  | Seita.3G270300 | peroxidase [EC:1.11.1.7] | down | 0 |
|  | Seita.3G347500 | peroxidase [EC:1.11.1.7] | down | 0 |
|  | Seita.4G047200 | shikimate O-hydroxycinnamoyltransferase [EC:2.3.1.133] | down | 0 |
|  | Seita.4G134400 | beta-glucosidase [EC:3.2.1.21] | down | 0 |
|  | Seita.4G135100 | peroxidase [EC:1.11.1.7] | down | 0 |
|  | Seita.5G035700 | cinnamoyl-CoA reductase [EC:1.2.1.44] | down | 0 |
|  | Seita.5G174100 | peroxidase [EC:1.11.1.7] | down | 0 |
|  | Seita.5G210200 | coniferyl-aldehyde dehydrogenase [EC:1.2.1.68] | down | 0 |
|  | Seita.5G210300 | coniferyl-aldehyde dehydrogenase [EC:1.2.1.68] | down | 0 |
|  | Seita.5G318800 | shikimate O-hydroxycinnamoyltransferase [EC:2.3.1.133] | down | 0 |
|  | Seita.6G026000 | cinnamyl-alcohol dehydrogenase [EC:1.1.1.195] | down | 0 |
|  | Seita.6G167900 | 4-coumarate--CoA ligase [EC:6.2.1.12] | down | 0 |
|  | Seita.7G029200 | cinnamyl-alcohol dehydrogenase [EC:1.1.1.195] | down | 0 |
|  | Seita.7G128200 | peroxidase [EC:1.11.1.7] | up | 0 |
|  | Seita.7G155700 | shikimate O-hydroxycinnamoyltransferase [EC:2.3.1.133] | down | 0 |
|  | Seita.7G168800 | phenylalanine ammonia-lyase [EC:4.3.1.24] | down | 0 |
|  | Seita.9G081400 | peroxidase [EC:1.11.1.7] | down | 0 |
|  | Seita.9G091400 | beta-glucosidase [EC:3.2.1.21] | up | 0 |
|  | Seita.9G184000 | 4-coumarate--CoA ligase [EC:6.2.1.12] | down | 0 |
|  | Seita.9G186200 | peroxidase [EC:1.11.1.7] | down | 0 |
|  | Seita.9G193900 | ferulate-5-hydroxylase [EC:1.14.-.-] | down | 0 |
|  | Seita.9G298200 | peroxidase [EC:1.11.1.7] | down | 0 |
|  | Setaria_italica_newGene_961 | peroxidase [EC:1.11.1.7] | down | 3.83419e-129 |

**The expression genes of ko00940 in LCKvsLT**

| **NO.** | **Gene ID** | **RefSeq** | **Expression** | **E value** |
| --- | --- | --- | --- | --- |
|  | Seita.4G247400 | peroxidase [EC:1.11.1.7] | down | 0 |
|  | Seita.4G176600 | peroxidase [EC:1.11.1.7] | up | 0 |
|  | Seita.2G199300 | cinnamyl-alcohol dehydrogenase [EC:1.1.1.195] | down | 0 |
|  | Seita.2G004800 | peroxidase [EC:1.11.1.7] | down | 0 |
|  | Seita.1G380000 | peroxidase [EC:1.11.1.7] | up | 0 |
|  | Seita.1G240600 | phenylalanine ammonia-lyase [EC:4.3.1.24] | down | 0 |
|  | Seita.1G240400 | phenylalanine ammonia-lyase [EC:4.3.1.24] | down | 0 |
|  | Seita.1G065800 | 4-coumarate--CoA ligase [EC:6.2.1.12] | down | 0 |
|  | Seita.1G023100 | peroxidase [EC:1.11.1.7] | up | 0 |
|  | Seita.5G046600 | peroxidase [EC:1.11.1.7] | up | 0 |
|  | Seita.5G361200 | trans-cinnamate 4-monooxygenase [EC:1.14.13.11] | down | 0 |
|  | Seita.7G128200 | peroxidase [EC:1.11.1.7] | up | 0 |
|  | Seita.7G155700 | shikimate O-hydroxycinnamoyltransferase [EC:2.3.1.133] | down | 0 |
|  | Seita.7G164500 | beta-glucosidase [EC:3.2.1.21] | up | 0 |
|  | Seita.7G168800 | phenylalanine ammonia-lyase [EC:4.3.1.24] | down | 0 |
|  | Seita.7G271100 | peroxidase [EC:1.11.1.7] | up | 0 |
|  | Seita.9G081300 | peroxidase [EC:1.11.1.7] | up | 0 |
|  | Seita.9G091400 | beta-glucosidase [EC:3.2.1.21] | up | 0 |
|  | Seita.9G193900 | ferulate-5-hydroxylase [EC:1.14.-.-] | down | 0 |
|  | Seita.9G537200 | 4-coumarate--CoA ligase [EC:6.2.1.12] | up | 0 |
|  | Seita.9G562600 | peroxidase [EC:1.11.1.7] | up | 0 |
|  | Seita.J003100 | peroxidase [EC:1.11.1.7] | up | 0 |
