# Supplementary material for: Integration of Metabolomics and Transcriptomics for Investigating the Tolerance of Foxtail Millet (Setaria italica) to Atrazine Stress
Source: Front Plant Sci. 2022 Jun 10;13:890550. doi: 10.3389/fpls.2022.890550 (PMC9226717; doi:10.3389/fpls.2022.890550)
Supplement: Supplementary file 14 [file Table_13.DOCX]

**Additional file 14. The expression of genes using qRT-PCR method.**

| **No.** | **Gene ID** | **Gene annotation** | **RNA-Seq (log2FC)** | | **qRT-PCR** |
| --- | --- | --- | --- | --- | --- |
|  |  |  | **GA** | **LG** |  |
| 1 | Seita.9G346100 | glutathione S-transferase GSTU6 [EC:2.5.1.18] | -1.98 | -- | 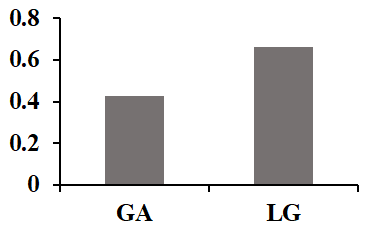 |
| 2 | Seita.9G345300 | glutathione S-transferase GSTU6 | -2.89 | -1.58 | 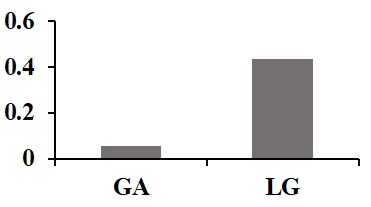 |
| 3 | Seita.5G328300 | glutathione S-transferase 1 | -1.06 | -0.65 | 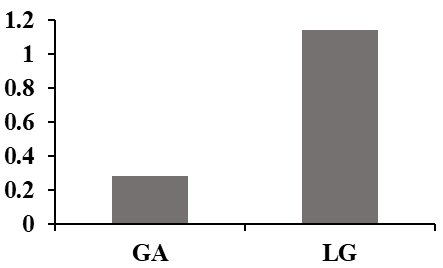 |
| 4 | Seita.5G452800 | glutathione transferase GST 23-like | -1.54 | -- | 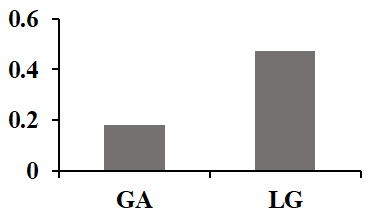 |
| 5 | Seita.8G008300 | glutathione transferase GST 23-like | -0.31 | -1.60 | 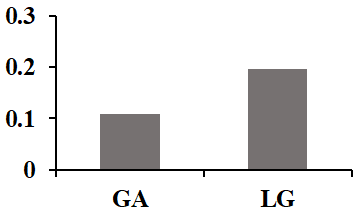 |
| 6 | Seita.4G241000 | glutathione transferase GST 23-like | -- | 1.68 | 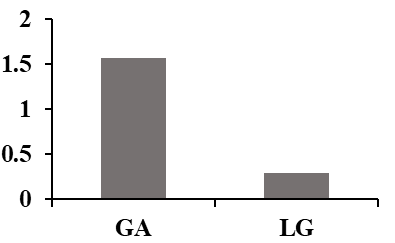 |
| 7 | Seita.5G453000 | glutathione S-transferase | -0.97 | -- | 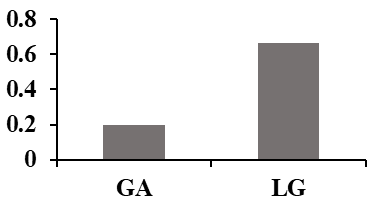 |
| 8 | Seita.9G424300 | glucose-6-phosphate 1-dehydrogenase 4 | -0.56 | -0.81 | 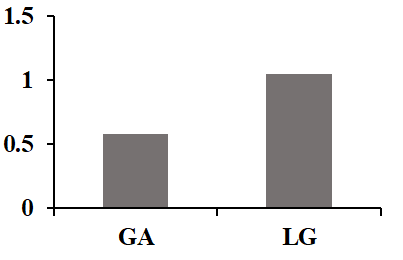 |
| 9 | Seita.5G442000 | glutathione S-transferase 3 | -0.20 | -0.19 | 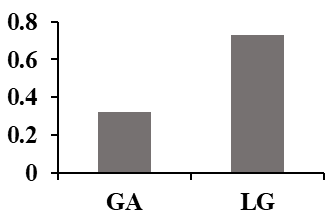 |
| 10 | Seita.3G038700 | glutathione S-transferase 4-like | -0.70 | -0.59 | 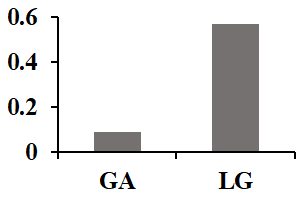 |
| 11 | Seita.3G038400 | glutathione S-transferase GSTF1 | -0.07 | -1.06 | 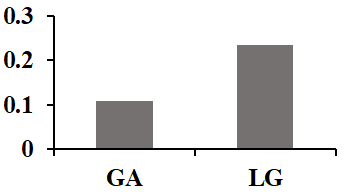 |
| 12 | Seita.9G121000 | microsomal glutathione S-transferase 3-like | -- | -1.13 | 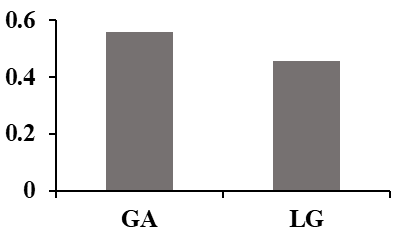 |
| 13 | Seita.3G194300 | coumaroylquinate(coumaroylshikimate) 3'-monooxygenase (*C3’H*) | 0.13 | -0.51 | 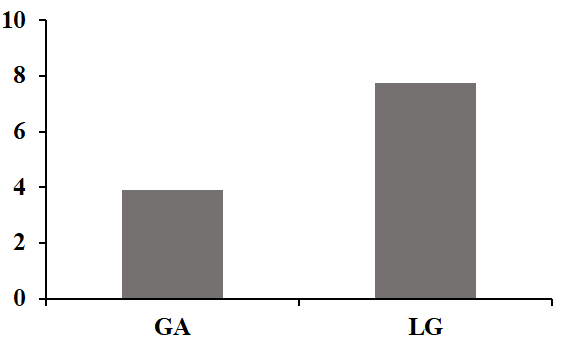 |
| 14 | Seita.4G047200 | shikimate O-hydroxycinnamoyl transferase-like (*HCT*) | 0.05 | -0.85 | 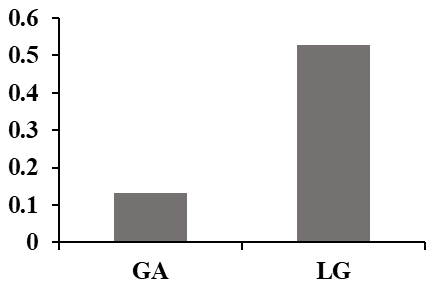 |
| 15 | Seita.3G024100 | glutamine synthetase | -0.61 | 1.88 | 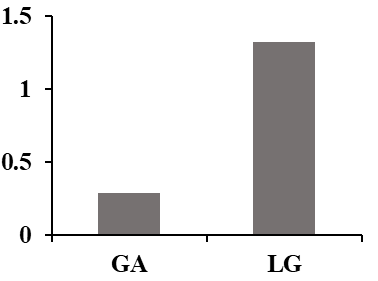 |
| 16 | Seita.8G075800 | acetolactate synthase small subunit 2 | -0.36 | -1.20 | 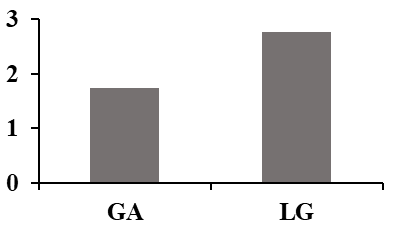 |
| 17 | Seita.9G118300 | glutamine synthetase cytosolic isozyme 1-3 | -- | 1.86 | 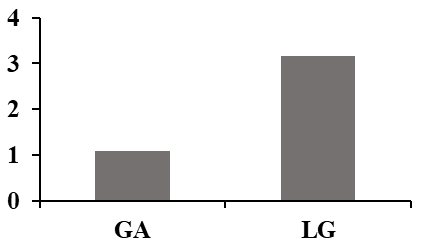 |
| 18 | Seita.5G381500 | transketolase, chloroplastic-like | -0.36 | 1.65 | 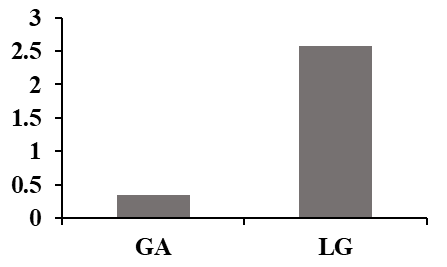 |
| 19 | Seita.7G188800 | acetylglutamate kinase-like | 0.37 | -1.49 | 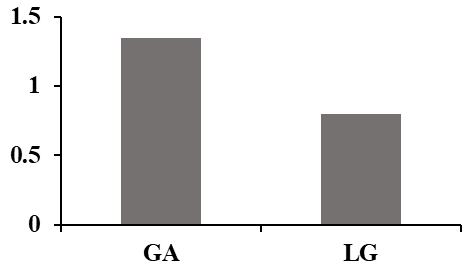 |
| 20 | Seita.1G147500 | 4-hydroxy-tetrahydrodipicolinate reductase 1 | 0.04 | -1.07 | 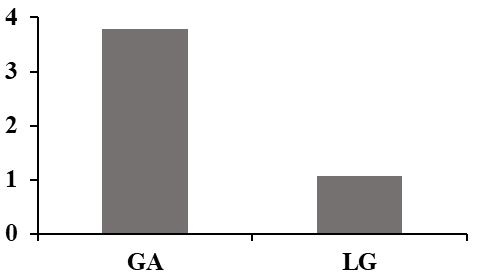 |
| 21 | Seita.1G292000 | histidinol-phosphate aminotransferase | -0.36 | 1.52 | 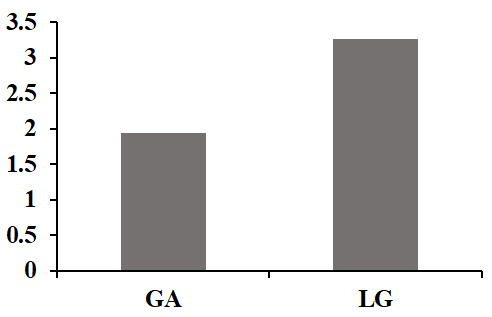 |
| 22 | Seita.9G534400 | phosphoserine aminotransferase 1 | 0.22 | -1.25 | 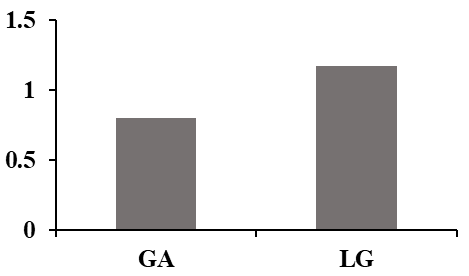 |
| 23 | Seita.7G265100 | D-3-phosphoglycerate dehydrogenase 3 | -0.30 | -1.27 | 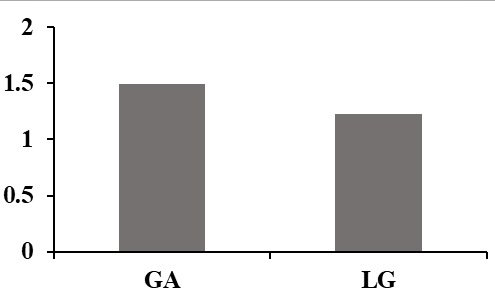 |
| 24 | Seita.9G010200 | aspartokinase 1 | -0.77 | 1.10 | 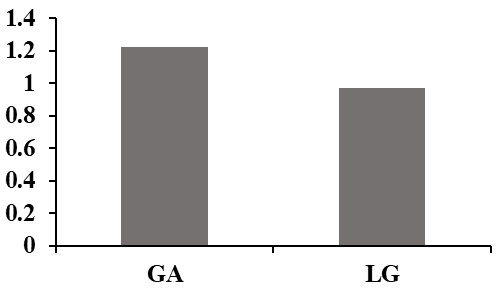 |
| 25 | Seita.7G046700 | ribose-5-phosphate isomerase 2 | -0.41 | 1.61 | 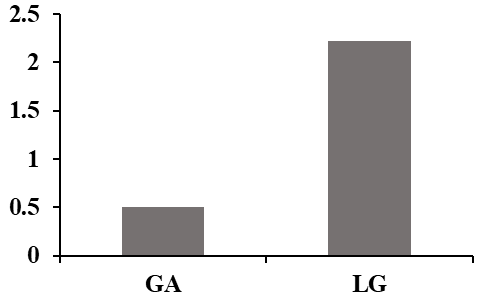 |
| 26 | Seita.5G272500 | glutamate synthase 1 | 0.45 | 1.09 | 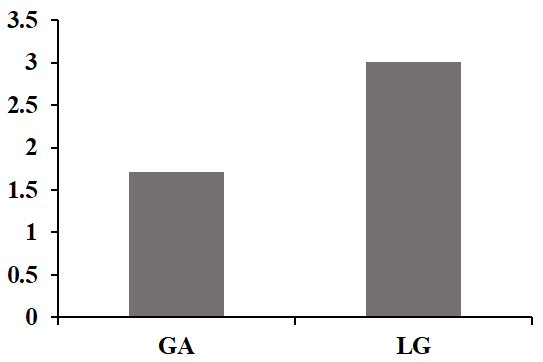 |
| 27 | Seita.7G164500 | beta-glucosidase 18 | -0.24 | 1.17 | 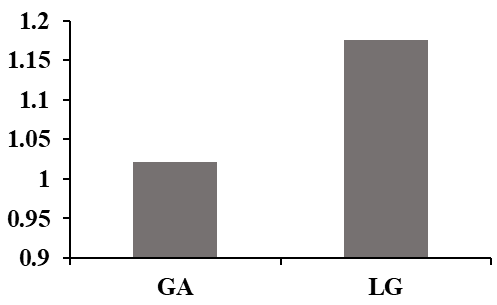 |
| 28 | Seita.1G023100 | peroxidase P7 | -0.57 | 1.24 | 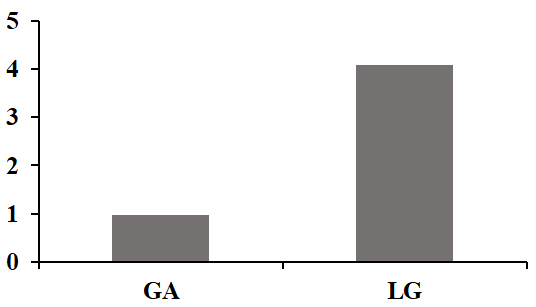 |
| 29 | Seita.7G271100 | peroxidase 5 | -0.83 | 1.31 | 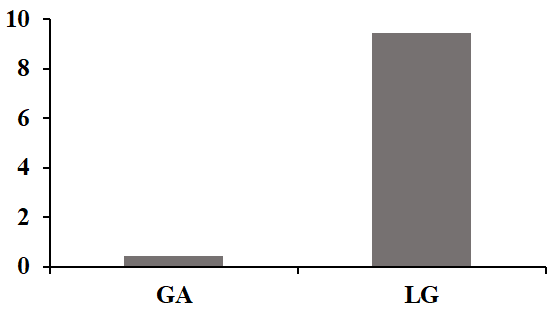 |
| 30 | Seita.J003100 | peroxidase 1 | -1.03 | 1.39 | 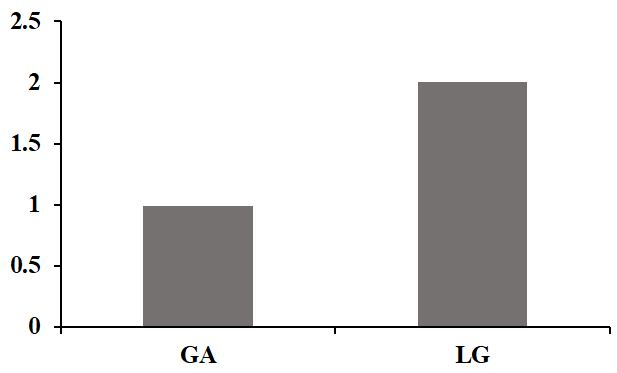 |
| 31 | Seita.7G128200 | peroxidase 31 | -0.40 | 1.45 | 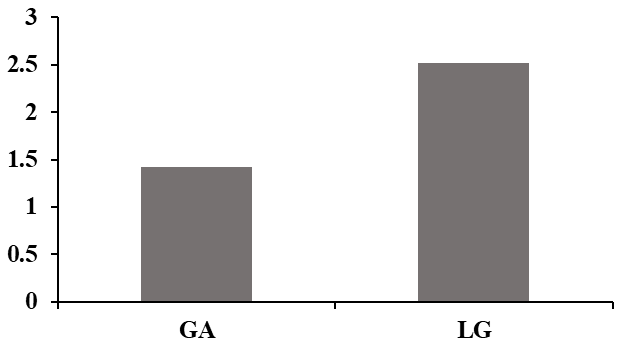 |
| 32 | Seita.9G562600 | peroxidase 5 | -0.62 | 1.16 | 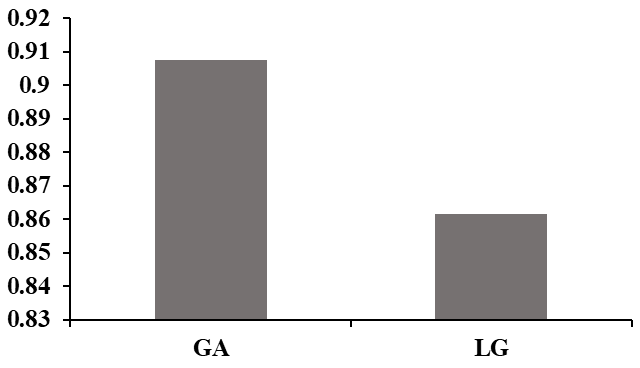 |
| 33 | Seita.6G036000 | lactoylglutathione lyase [EC:4.4.1.5] | 0.92 | -1.05 | 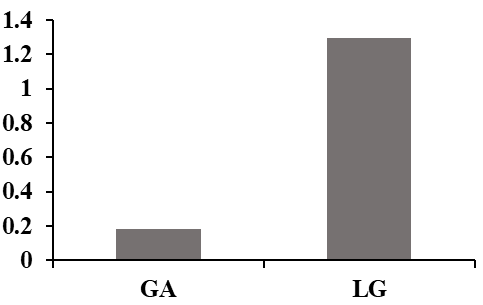 |
| 34 | Seita.3G096900 | glutathione S-transferase | -0.45 | -0.59 | 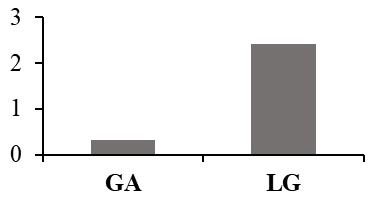 |

Note: -- means no change
